# Assessing the prevalence, characteristics and psychosocial correlates of nonsuicidal self-injury among Vietnamese adolescent psychiatric outpatients: a cross-sectional study

**DOI:** 10.3389/fpsyt.2026.1699844

**Published:** 2026-02-18

**Authors:** Hoang Yen Nguyen, Xuan Thang Pham, Cong Thien Le, Thi Thu Ha Le, Thi Thu Ha Tran, Thi Hue Doan, Thien Thang Tran, Tuan Khiem Ngo, Thi Nguyet Nga Pham, Thi Ha An Tran, Van Tuan Nguyen

**Affiliations:** 1 Department of Psychiatry, Hanoi Medical University, Hanoi, Vietnam; 2 National Institute of Mental Health, Bach Mai Hospital, Hanoi, Vietnam; 3 Department of Psychiatry, Faculty of Medicine, Can Tho University of Medicine and Pharmacy, Can Tho, Vietnam

**Keywords:** DSM-5, nonsuicidal self-injury, NSSI, self-harm, Vietnam

## Abstract

**Background:**

Nonsuicidal self-injury (NSSI) is a global public health concern due to its significant negative impact on adolescents’ mental health and well-being. However, data on NSSI among Vietnamese youth remain limited. This study examined the prevalence, characteristics, and correlates of NSSI among Vietnamese adolescents seeking mental health support.

**Method:**

A cross-sectional study was conducted on adolescents aged 10–19 years attending outpatient departments at three psychiatric centers in Hanoi from May to November 2024. Participants were interviewed face-to-face by senior psychiatrists, and NSSI diagnosis was assessed using DSM-5-TR criteria (NSSI DSM-5). Univariable and multivariable logistic regression identified associated factors.

**Results:**

The 12-month prevalence of participants who met criteria for NSSI DSM-5 was 17.9%. Females reported a higher prevalence of NSSI DSM-5 and a greater urge to self-injure than males. In crude analyses, females also reported a greater diversity of NSSI methods and more body areas affected; however, these differences were not significant after adjustment. Scratching, cutting, and biting were the most common methods; cutting was more prevalent among females. NSSI motives were often combined, with the main purposes being relief from negative emotions or cognitive states and the induction of positive feelings, rather than coping with interpersonal difficulties. Shared factors associated with NSSI in both genders included a family history of self-harm, emotional abuse, and higher McLean Screening Instrument for Borderline Personality Disorder scores.

**Conclusion:**

These findings raise awareness of NSSI among Vietnamese psychiatrists and provide important data for a low- and middle-income country with unique cultural characteristics. Further research and culturally sensitive interventions are needed.

## 1 Introduction

Nonsuicidal self-injury (NSSI) refers to the deliberate, self-inflicted damage of body tissue without suicidal intent and for reasons that are not socially sanctioned, such as cutting, burning, or scratching oneself (1). Although NSSI involves physical harm, its primary function is often to help individuals cope with intense negative emotions or manage psychological distress, rather than to escape unbearable emotional pain associated with suicidal behavior (2). NSSI has emerged as a significant public health concern, particularly among children and adolescents, due to its adverse effects on health and overall quality of life (2, 3). Recent research indicates that the severity and frequency of NSSI behaviors are increasing, with adolescents in modern society displaying a greater tendency to engage in repetitive acts of self-injury, including more moderate and severe forms over time (4). This pattern suggests not only immediate physical harm but also long-term cumulative health consequences. Longitudinal studies have shown that individuals who engage in NSSI during early adolescence experience higher levels of stress, anxiety, continued self-injury, and difficulties with emotion regulation even a decade later (3). Moreover, recurrent NSSI is a predictor of future psychiatric disorders, including depression and eating disorders (5). While there is a clear distinction between NSSI and suicidal attempts, NSSI remains one of the strongest risk factors for suicide, with individuals who self-injure facing up to a 30-fold increased risk (6).

While NSSI is a widespread phenomenon, its prevalence rates vary considerably across studies. Estimates range from 7.5% to 46.5% among adolescents in Western populations (2), 7.1% to 11.4% in community samples and 20.7% to 75.9% in clinical samples within Southeast Asian countries (7), and 5% to 16.4% in clinical populations in South Asia (8). These variations largely result from differences in the criteria or definitions of NSSI used across studies (2). Historically, NSSI was viewed primarily as a symptom of other mental disorders, such as borderline personality disorder (BPD). However, accumulating research over recent decades has demonstrated that NSSI has distinct prevalence, clinical features, trajectories, and outcomes, independent of other mental health conditions (2, 9). In recognition of its clinical significance, DSM-5 (10) was the first to include non-suicidal self-injury (NSSI), listing it in Section III (“Conditions for Further Study”), then in Section II “Other conditions that may be a Focus of Clinical Attention” in DSM-5-TR (11). NSSI was also included in ICD-11, section “Symptoms, signs or clinical findings, not elsewhere classified” (12). These inclusion reflect the field’s acknowledgment of NSSI’s importance, while also emphasizing the need for further empirical validation before it can be classified as a formal diagnosis. Consequently, there is an urgent need for a comprehensive understanding of NSSI’s characteristics and underlying factors to inform the development of effective interventions.

In the Vietnamese context, NSSI is emerging as a significant issue among adolescents, with up to 43.9% of high school students aged 15–18 years reporting engagement in at least one type of NSSI within the previous 12 months (13). Clinically, NSSI is commonly encountered by psychiatrists in Vietnam. However, the ICD-10 classification of mental and behavioral disorders, which is officially used in Vietnam, identifies NSSI only as a symptom of BPD and does not recognize it as an independent disorder entity (14). As a result, patients whose primary manifestation is NSSI are often categorized under “F92: mixed disorders of conduct and emotions” in clinical practice. This theoretical gap poses challenges for the effective diagnosis and treatment of NSSI in Vietnam. To address this issue, we investigated the prevalence, characteristics, functions, and correlates of NSSI diagnosed using DSM-5-TR criteria (hereafter “NSSI DSM-5”) in outpatient psychiatric settings, distinguishing it from NSSI defined by other diagnostic systems and from questionnaire-based assessments. DSM-5-TR (Section II) proposes NSSI as intentional self-inflicted injury without suicidal intent occurring on ≥5 days in the past year, carried out to regulate internal states (e.g., relieve negative affect) or address interpersonal distress, and associated with distress/impairment. The behavior must be non–socially sanctioned and not better explained by psychosis, intoxication/withdrawal, neurodevelopmental stereotypies, or another mental/medical condition. Our aim is to provide initial data for Vietnamese psychiatrists and to contribute local insights to the global understanding of NSSI. The findings from this study will also inform future research and consensus-building efforts on this important topic.

## 2 Methods

### 2.1 Study design and settings

This study used a cross-sectional design conducted from May 2024 to November 2024 at the outpatient department of three medical institutions in Hanoi – the capital of Vietnam: (1) National Institute of Mental health (NIMH), (2) Hanoi Psychiatric hospital (HPH), and (3) Mental health division of Hanoi Medical University Hospital (HMUH). NIMH is a national-level psychiatric institution that is responsible for the mental health care of Northern Vietnam; HPH is the provincial-level psychiatric hospital, and HMUH is one of the biggest general hospitals in Hanoi, Vietnam.

### 2.2 Participants and sampling

All patients aged 10–19 presenting at the outpatient department of the three institutions were initial potentially eligible participants for the study. After being invited and explained the relevant information of study, if agreed to participate, the participant signed an informed consent under the supervision of their guardian. Those (1) who did not have legal guardian at the time of presentation or (2) who and/or their guardian disagreed with participating in or (3) who had limited ability to communicate with physicians or comprehend the questions or (4) who did not complete the questionnaire were excluded from the study.

**Figure 1:**
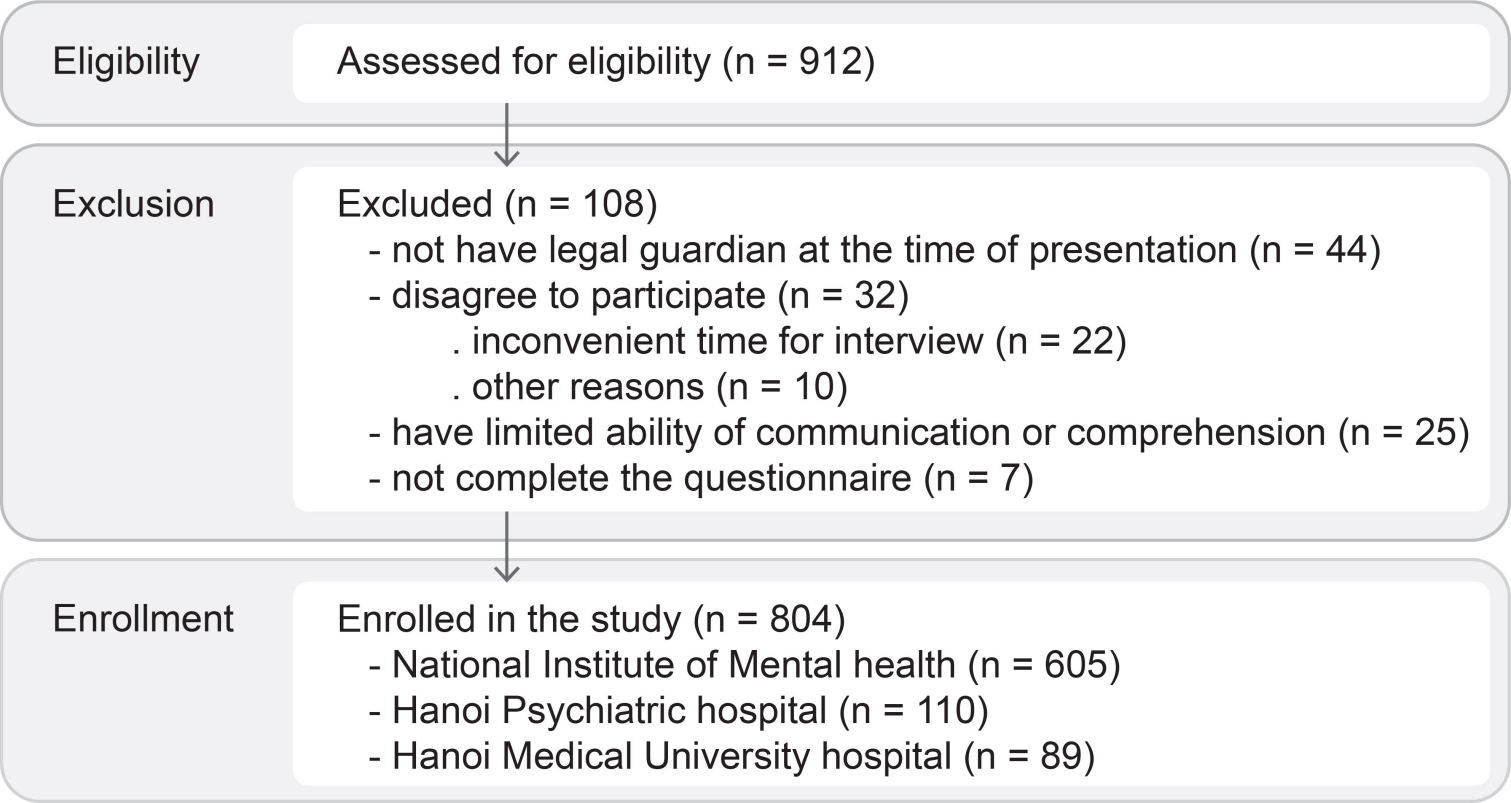
Consort diagram of the participants recruitment.

A convenient sampling technique was implemented, and the participants’ anonymity and confidentiality were guaranteed. To ensure the sample representativeness and the power of statistics, we calculated the minimum sample size using G*Power (version 3.1.9.7) (15) with a conservative estimate of proportion of 0.5, a confidence level of 95%, and a margin of error of 5%. The calculated result was 385. The recruitment process is summarized in **Figure 1**.

### 2.3 Data collection

The participants underwent the standard clinical examination at the outpatient department. After agreeing to participate in the study, the patients continued to be assessed to determine whether or not to be fulfilled the criteria of NSSI DSM-5. This clinical diagnosis was implemented by the psychiatrists from the Department of Psychiatry, Hanoi Medical University, and were core members of the research team. Finally, the participants continued to answer a developed questionnaire under the instructions/supervision of well-trained researchers. The required duration to complete the questionnaire was roughly 40–50 minutes.

### 2.4 Variables and measurements

A questionnaire, including items designed to assess NSSI DSM-5, was developed to collect detailed information. Interviewers received standardized training and conducted a pilot study with 20 outpatient adolescents prior to data collection. During the pilot, participants’ understanding and cognitive processing of the items were evaluated through face-to-face assessment to establish face validity, and ambiguous or potentially controversial wording was revised as needed to improve clarity and acceptability. The pilot phase also served to calibrate interviewers’ application of the NSSI DSM-5 assessment, with consensus discussions used to standardize ratings and strengthen inter-rater reliability.

#### 2.4.1 NSSI according to DSM-5-TR

The English DSM-5-TR criteria for NSSI were translated into Vietnamese. Psychiatrists were received training to ensure a thorough and consistent understanding and application of the DSM-5-TR criteria. To enhance inter-rater reliability for NSSI DSM-5 diagnosis, we developed a semi-structured questionnaire based on the DSM-5-TR criteria from A–F; criterion F also required additional clinical information. The questionnaire included open-ended questions to elicit initial information, followed by closed questions as needed to confirm specific details. Detailed information on the questionnaire is provided in **Supplementary Material 1**.

#### 2.4.2 Functions of NSSI

The functions of NSSI were categorized into four groups: *automatic negative reinforcement, automatic positive reinforcement, social negative reinforcement*, and *social positive reinforcement*, based on the framework proposed by Nock and Prinstein (16). The specific manifestations of each group were informed in part by the Functional Assessment of Self-Mutilation (FASM) questionnaire (17). Using the FASM as a primary source for item content, we operationalized NSSI function manifestations and mapped them to the four Nock and Prinstein function categories. To empirically confirm this mapping and support the proposed four-factor structure, we performed confirmatory factor analysis (CFA). More details are available in the **Supplementary Material 2**.

#### 2.4.3 Other information

Socio-demographic characteristics encompassed *gender*, *age*, *educational background*, *residential area*, and *family financial status*. Other psychological and clinical characteristics included: *quality of parents’ relationship* and *parents–participant (child) relationship*, *history of self-harm behaviors in the family*, *having significant stressful event(s) recently*, *history of substance use in the recent three months.* The participants were also assessed for their *childhood trauma experiences* using the Childhood Trauma Questionnaire – Short Form (CTQ–SF) (18), *borderline personality disorder risk* using the McLean Screening Instrument for BPD (MSI–BPD) (19), and *urge to self-injure* using the Alexian Brothers Urge to Self-Injure Scale (ABUSI) (20). The English questionnaire was forward-translated into Vietnamese by two independent translators (a psychiatrist and a non-medical professional). The Vietnamese version was then back-translated into English by two additional independent translators (a psychiatrist and a non-medical professional) to assess equivalence with the original instrument. Discrepancies were resolved by consensus among the translators and the principal investigators. Subsequent analyses included content validity index ratings by two experts (a psychiatrist and a psychologist), inter-item correlations, CFA, measurement invariance testing across subgroups (gender and age), and Cronbach’s α to evaluate internal consistency.

- CTQ–SF, a 28-item (25 clinical items and three validity items) retrospective, self-reported questionnaire, shows as a well-adapted, reliable, and valid tool to measure five traumatic experience domains in childhood, including physical, emotional, and sexual abuse and physical and emotional neglect (18, 21, 22). Each item’s response is quantified on a 5-point Likert scale (1 = *never true*, 2 = *rarely true*, 3 = *sometimes true*, 4 = *often true*, and 5 = *very often true*). Each domain has 5 items, and its total score ranges from 5 to 25. The higher score indicates a higher level of personal traumatic experience in childhood. With the exception of the physical neglect subscale, which was excluded due to low internal consistency (Cronbach’s α = 0.47), the remaining subscales demonstrated good reliability and were included in the analyses. Detailed validation results are provided in **Supplementary Material 3**.
- MSI–BPD is a 10-item self-reported instrument used to screen for the risk of BPD. Each item is scored dichotomously (yes = 1, no = 0), yielding a total score ranging from 0 to 10; higher scores indicate a greater likelihood of BPD. Supporting materials are provided in **Supplementary Material 4**.
- ABUSI is a questionnaire created to measure the degree of urge to self-injure under both cognitive and emotional aspects in the last week. This questionnaire encompasses five seven-point Likert-scale questions (0 – 6), and the sum score ranges from 0 to 30, which higher score reflect higher urge. The cut-off point of 4 is set to discriminate between subjects with a higher probability of NSSI DSM-5 in this study. Detailed validation results are provided in **Supplementary Material 5**.

### 2.5 Statistical analysis

Data analysis and visualization were conducted using R version 4.5. Initially, exploratory and descriptive analyses were performed to summarize the sample characteristics, reporting means, standard deviations, frequencies, and percentages. Subsequently, bivariate analyses — including the Wilcoxon rank sum test, Pearson’s Chi-squared test, and Fisher’s exact test — were used with false discovery rate (fdr) correction where applicable to compare differences between groups.

We performed logistic regression analyses to identify factors associated with NSSI DSM-5, stratified by gender. Candidate predictors were pre-specified *a priori* based on the conceptual framework and prior literature and were grouped into domains (sociodemographic characteristics, family/living conditions, personal psychological factors, comorbid psychiatric disorders, and other factors). First, all variables were examined using univariable logistic regression and reported as odds ratios (ORs) with 95% confidence intervals (CIs). For the multivariable analyses, all candidate variables were initially entered into the multivariable model. To account for potential between-center differences, “Institution” variable was also included in all multivariable models as a categorical fixed effect (indicator variables). Multicollinearity was then assessed using the variance inflation factor (VIF). When multicollinearity was identified (VIF > 3), we inspected correlated variables within the same domain and removed redundant variables, prioritizing retention of the most clinically interpretable and conceptually representative indicator. The model was refitted iteratively, with VIF and goodness-of-fit reassessed after each revision, until a stable model with acceptable diagnostics was obtained. Model fit was assessed using the Hosmer–Lemeshow goodness-of-fit test. Detailed information is provided in **Supplementary Material 6**.

All p-values were two-sided, and p < 0.05 was considered statistically significant.

## 3 Results

**Figure 1** presents the recruitment process. In this study, 912 participants aged 10–19 years were eligible for enrollment. Among the 108 excluded participants, 22 declined to participate due to inconvenient scheduling (late interviews and limited time for commuting home). An additional 7 participants did not complete the questionnaire for similar reasons. However, 10 participants declined participation without providing a reason. The final recruited number in this study was 804. The main sociodemographic characteristics of participants and their psychiatric diagnoses are summarized in **Table 1**. Almost all patients (91.3%) did not have a previous mental health diagnosis.

**Table 1:** Sociodemographic characteristics and classification of primary psychiatric diagnosis of participants.

Variable	N = 804
Gender	
Male	345 (42.9%)
Female	459 (57.1%)
**Age (years)**	15.25 (2.1)
Educational level	
< High school	323 (40.2%)
High school and above	481 (59.8%)
Living area	
Urban	510 (63.4%)
Rural	294 (36.6%)
Family income	
≤ 10 million VND	417 (51.9%)
> 10 million VND	387 (48.1%)
Living with	
Parents	667 (83.0%)
Father or Mother	92 (11.4%)
Others	45 (5.6%)
**Puberty** (Yes)	731 (90.9%)
Sexual orientation	
Heterosexual	469 (58.3%)
Non-heterosexual	69 (8.6%)
Unknown/Unanswered	266 (33.1%)
**History of having psychiatric diagnosis** (Yes)	70 (8.7%)
**Required hospitalization** (Yes)	144 (17.9%)
Primary (main) clinical diagnosis	
Mixed disorders of conduct and emotions	309 (42.1%)
Anxiety disorder	92 (12.5%)
Depressive disorder	91 (12.4%)
Reaction to severe stress, and adjustment disorders	41 (5.6%)
Acute and Transient Psychotic Disorders	22 (3.0%)
Hyperkinetic disorders	21 (2.9%)
Sleep disorders not due to a substance or known physiological condition	20 (2.7%)
Dissociative (conversion) disorders	15 (2.0%)
Bipolar spectrum disorder	10 (1.4%)
Others	113 (15.4%)
Not fulfill any clinical diagnosis	70 (8.7%)

n (%), Mean (SD).

Bold values indicate statistically significant results.

**Figure 2:**
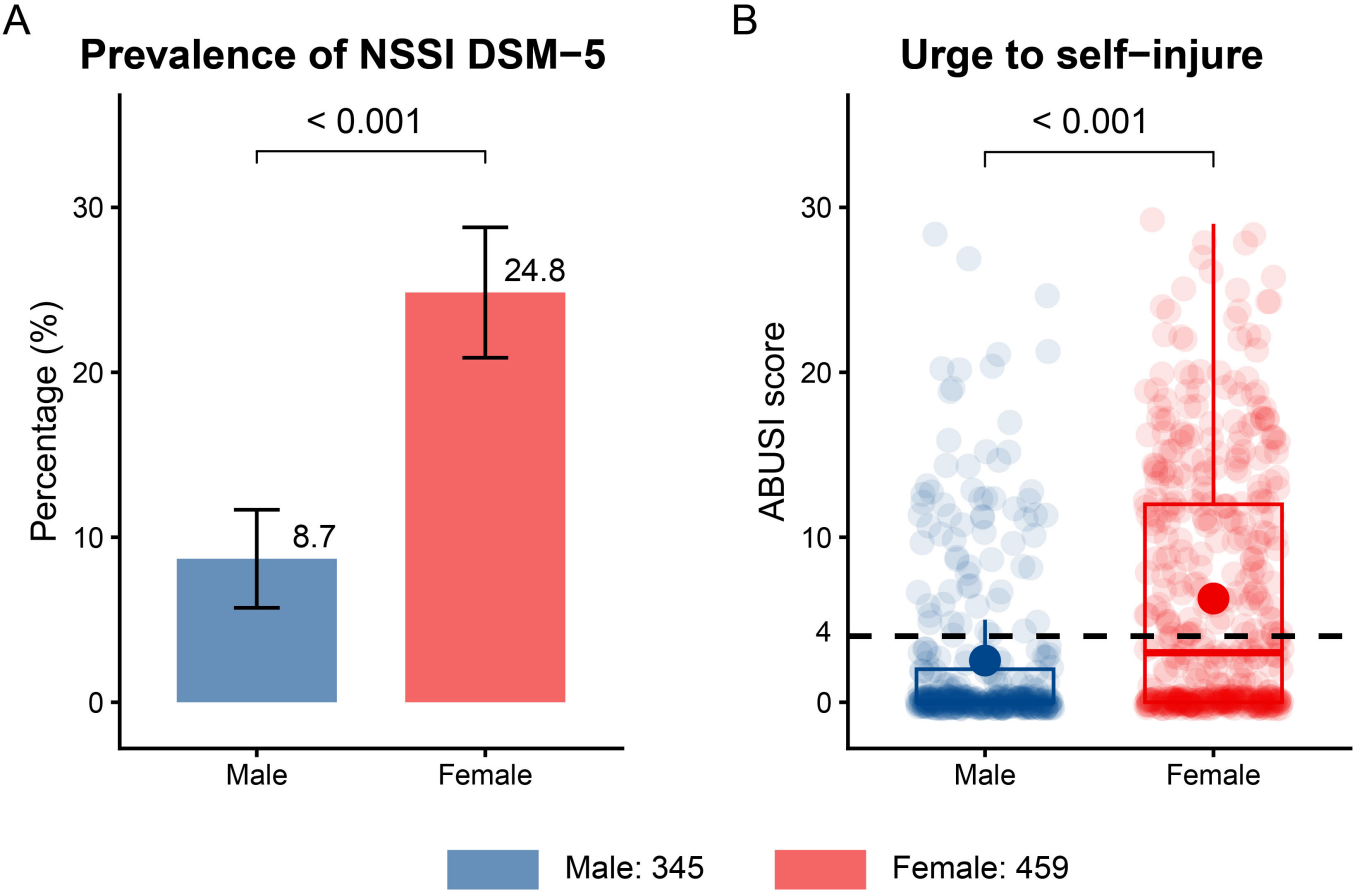
The 12-month prevalence of NSSI DSM-5 between gender and the current urge to self-injure measured by ABUSI questionnaire in the sample. **(A)** The frequency of NSSI among each gender, error bars indicate 95% confidence interval; difference between genders were evaluated using the *Pearson’s Chi-Square test*. **(B)** The data are presented as boxplots and jitter plots; dark points are mean values of group; dashed line is the threshold of high probability of NSSI DSM5. Difference between genders were evaluated using the *Wilcoxon rank sum test.*

**Figure 2** presented the NSSI DSM-5 and urge to self-injure status. Overall, 144 (17.9%) participants met the criteria for NSSI DSM-5, and females demonstrated a higher 12-month prevalence of NSSI DSM-5 (24.8%) compared to males (8.7%) (*χ^2^* = 33.812, *p* < 0.001) (**Figure 2A**). At the time of examination, female patients exhibited significantly higher scores in the ABUSI questionnaire than male patients (W = 54,053, *p* < 0.001), indicating a greater current urge to self-injure in the female group (**Figure 2B**).

**Figure 3:**
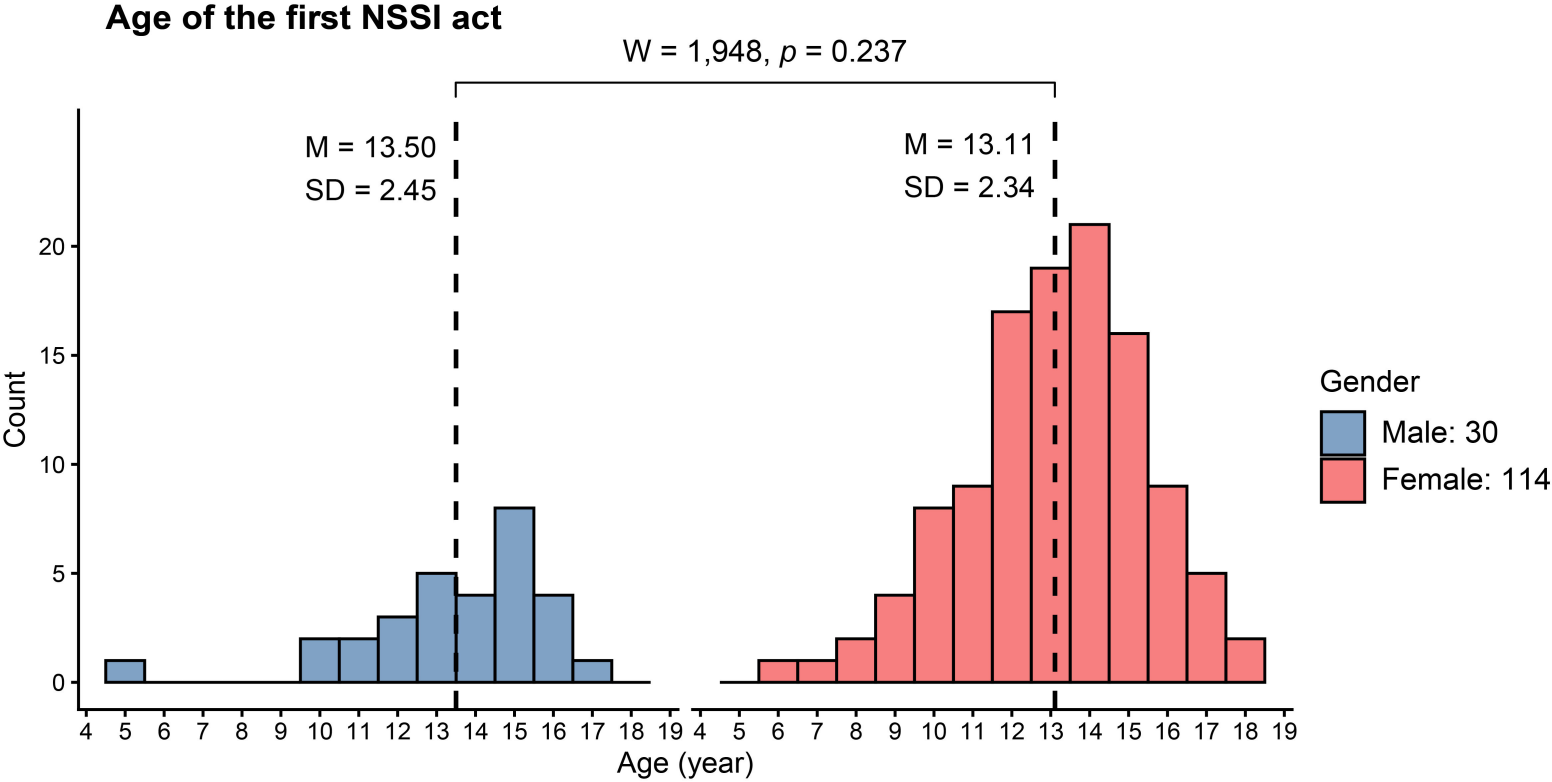
Histogram presenting the age of the first NSSI act by gender. Dashed vertical lines represent for means. Comparison between means was performed using the *Wilcoxon rank sum test.*

**Figure 3** indicates that the first onset of NSSI behaviors predominantly occurred between the ages of 12 and 15 years in females (mean 13.11 ± 2.34 years) and between 12 and 16 years in males (mean 13.50 ± 2.45 years), with no significant difference between genders (W = 1,948, *p* = 0.237). **Table 2** summarizes the characteristics of NSSI behaviors in this cohort. Scratching and cutting were the most common methods of deliberate self-injury among both genders. Other methods, including biting, wound picking, preventing wounds from healing, and hitting, were also reported but occurred less frequently. Cutting was more prevalent among female patients and remained significant after correction, while hitting and swallowing sharp objects were numerically more common among males but did not remain significant after adjustment. Female patients reported a higher number of NSSI methods than males (W = 1,302; *p* = 0.037), but the difference was not significant after adjustment (*q* = 0.18). The arm, forearm, and wrist were the most targeted anatomical sites for NSSI acts. Importantly, nearly all patients reported engaging in these behaviors alone and without the awareness of others.

**Table 2:** Characteristics of NSSI acts among NSSI DSM-5 patients.

Characteristic	Overall N = 144	Male N = 30	Female N = 114	OR (95% CI)	p-value*^1^*	q-value*^2^*
Forms of NSSI act						
Scratching	100 (69.4%)	17 (56.7%)	83 (72.8%)	2.04 (0.81 - 5.06)	0.088	0.32
Cutting	93 (64.6%)	12 (40.0%)	81 (71.1%)	3.64 (1.48 - 9.32)	**0.002**	**0.023**
Biting	40 (27.8%)	6 (20.0%)	34 (29.8%)	1.69 (0.60 - 5.53)	0.29	0.64
Wound picking	32 (22.2%)	7 (23.3%)	25 (21.9%)	0.92 (0.33 - 2.85)	0.87	>0.99
Preventing wounds from healing	24 (16.7%)	5 (16.7%)	19 (16.7%)	1.00 (0.32 - 3.77)	>0.99	>0.99
Hitting	19 (13.2%)	8 (26.7%)	11 (9.6%)	0.30 (0.10 - 0.95)	**0.029**	0.18
Skin carving	11 (7.6%)	0 (0.0%)	11 (9.6%)		0.12	0.35
Burning	10 (6.9%)	0 (0.0%)	10 (8.8%)		0.12	0.35
Sticking/Inserting sharp objects into the skin	7 (4.9%)	1 (3.3%)	6 (5.3%)	1.61 (0.18 - 76.60)	>0.99	>0.99
Self bone-breaking	6 (4.2%)	2 (6.7%)	4 (3.5%)	0.51 (0.07 - 5.93)	0.60	0.92
Swallowing sharp objects	4 (2.8%)	3 (10.0%)	1 (0.9%)	0.08 (0.00 - 1.06)	**0.029**	0.18
**Number of methods**	2.40 (1.5)	2.03 (1.4)	2.50 (1.5)		**0.037**	0.18
Body positions of NSSI act						
Arm/Forearm	80 (55.6%)	8 (26.7%)	72 (63.2%)	4.66 (1.80 - 13.25)	**<0.001**	**0.010**
Wrist	61 (42.4%)	11 (36.7%)	50 (43.9%)	1.35 (0.55 - 3.44)	0.48	0.87
Thigh	34 (23.6%)	4 (13.3%)	30 (26.3%)	2.31 (0.71 - 9.86)	0.14	0.36
Head	29 (20.1%)	6 (20.0%)	23 (20.2%)	1.01 (0.35 - 3.38)	0.98	>0.99
Finger	24 (16.7%)	5 (16.7%)	19 (16.7%)	1.00 (0.32 - 3.77)	>0.99	>0.99
Belly/Chest	16 (11.1%)	3 (10.0%)	13 (11.4%)	1.16 (0.29 - 6.78)	>0.99	>0.99
Face	15 (10.4%)	6 (20.0%)	9 (7.9%)	0.35 (0.10 - 1.30)	0.086	0.32
Lower leg	10 (6.9%)	1 (3.3%)	9 (7.9%)		0.69	>0.99
Other parts	9 (6.3%)	3 (10.0%)	6 (5.3%)	0.50 (0.10 - 3.30)	0.39	0.76
Foot	3 (2.1%)	0 (0.0%)	3 (2.6%)		>0.99	>0.99
**Number of positions**	1.95 (1.2)	1.57 (0.9)	2.05 (1.3)		**0.036**	0.18
Environmental situation during NSSI act						
Alone	132 (91.7%)	26 (86.7%)	106 (93.0%)	2.03 (0.41 - 8.30)	0.27	0.64
When relatives around	16 (11.1%)	5 (16.7%)	11 (9.6%)	0.54 (0.15 - 2.15)	0.33	0.68
When friends around	5 (3.5%)	0 (0.0%)	5 (4.4%)		0.58	0.92
When strangers around	4 (2.8%)	0 (0.0%)	4 (3.5%)		0.58	0.92

n (%), mean (SD).

*^1^*Pearson’s Chi-squared test; Fisher’s exact test; Wilcoxon rank sum test.

*^2^*False discovery rate correction for multiple testing.

CI, Confidence Interval; OR, Odds Ratio.

Bold values indicate statistically significant results.

**Figure 4:**
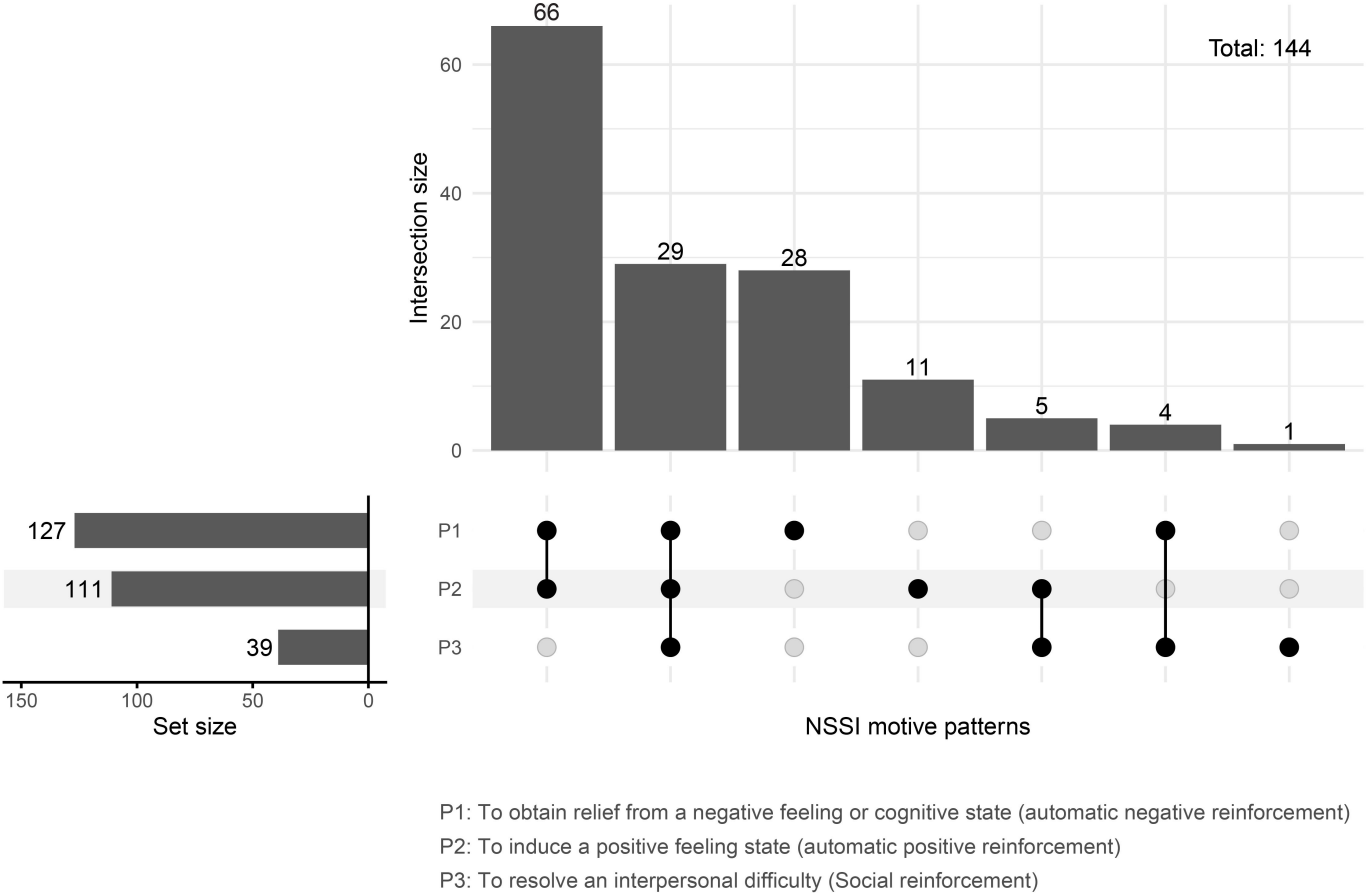
Upset plot illustrating the patterns of NSSI motives.

**Table 3** presents the distribution of the purposes underlying NSSI acts among patients meeting for NSSI DSM-5. Overall, there was no significant difference in motive between genders, except for the motive “to relieve feeling “numb” or empty.” The reported purposes were varied and complex, with “to stop bad feelings”, “to feel relaxed”, “to deal with anger”, and “to deal with disappointment” among the most frequently endorsed reasons. **Figure 4** (UpSet plot) further explored the patterns of NSSI motives as defined by diagnostic criteria. The most prevalent motives were obtaining relief from a negative emotional or cognitive state (P1, 127/144; 88.2%) and inducing a positive feeling state (P2, 111/144; 77.1%). In contrast, self-injury intended to resolve an interpersonal difficulty (P3, 39/144; 27.1%) was less commonly reported. Notably, most participants engaged in NSSI for a combination of motives rather than a single reason. Specifically, the P1-P2 combination accounted for 45.8% (66/144) of cases, while the presence of all three motives (P1-P2-P3) was observed in 20.1% (29/144) of participants. Only 20.0%, 7.6%, and 0.7% of participants reported engaging in NSSI solely for P1, P2, and P3, respectively.

**Table 3:** Purposes of NSSI acts among NSSI DSM-5 patients.

Characteristic	Overall N = 144	Male N = 30	Female N = 114	OR (95% CI)	p-value*^1^*	q-value*^2^*
Automatic negative reinforcement						
To stop bad feelings	98 (68.1%)	17 (56.7%)	81 (71.1%)	1.87 (0.74 - 4.62)	0.13	0.64
To deal with anger	72 (50.0%)	15 (50.0%)	57 (50.0%)	1.00 (0.41 - 2.42)	>0.99	>0.99
To deal with disappointment	70 (48.6%)	12 (40.0%)	58 (50.9%)	1.55 (0.64 - 3.88)	0.29	0.64
To relieve feeling “numb” or empty	62 (43.1%)	6 (20.0%)	56 (49.1%)	3.83 (1.39 - 12.33)	**0.004**	**0.048**
To avoid suicidal thoughts	40 (27.8%)	10 (33.3%)	30 (26.3%)	0.72 (0.28 - 1.92)	0.45	0.64
Automatic positive reinforcement						
To feel relaxed	75 (52.1%)	13 (43.3%)	62 (54.4%)	1.55 (0.64 - 3.84)	0.28	0.64
To feel something, even if it was pain	57 (39.6%)	10 (33.3%)	47 (41.2%)	1.40 (0.56 - 3.67)	0.43	0.64
To punish self	55 (38.2%)	9 (30.0%)	46 (40.4%)	1.57 (0.62 - 4.27)	0.30	0.64
To have a feeling of full control of self and life	34 (23.6%)	6 (20.0%)	28 (24.6%)	1.30 (0.46 - 4.29)	0.60	0.71
Social positive reinforcement						
To get more care from parents or friends	27 (18.8%)	3 (10.0%)	24 (21.1%)	2.39 (0.65 - 13.33)	0.17	0.64
To get attention	17 (11.8%)	4 (13.3%)	13 (11.4%)	0.84 (0.23 - 3.82)	0.75	0.82
To manipulate others	5 (3.5%)	0 (0.0%)	5 (4.4%)		0.58	0.71
Social negative reinforcement						
To avoid school, work or any social interaction,	10 (6.9%)	3 (10.0%)	7 (6.1%)	0.59 (0.12 - 3.77)	0.43	0.64

n (%).

*^1^*Pearson’s Chi-squared test; Fisher’s exact test.

*^2^*False discovery rate correction for multiple testing.

CI, Confidence Interval; OR, Odds Ratio.

Bold values indicate statistically significant results.

**Table 4** presents gender-stratified logistic regression analyses of factors associated with NSSI DSM-5 risk. In univariable models, several sociodemographic, family, clinical, and psychosocial variables were associated with NSSI DSM-5 risk. Multivariable models were then fitted. The models demonstrated good fit, as indicated by the Hosmer–Lemeshow goodness-of-fit test (females: χ² = 10.85, df = 8, *p* = 0.21; males: χ² = 4.43, df = 8, *p* = 0.816). Across both genders, three factors consistently remained associated with higher NSSI DSM-5 risk after adjustment: family history of self-harm (female: OR = 4.41 [2.48–7.95], *p* < 0.001; male: OR = 3.15 [1.13–10.5], *p* = 0.042), greater childhood emotional abuse (CTQ-SF emotional abuse; female: OR = 1.09 [1.02–1.17], *p* = 0.014; male: OR = 1.26 [1.08–1.49], *p* = 0.004), and higher MSI–BPD scores (female: OR = 1.17 [1.06–1.30], *p* = 0.003; male: OR = 1.27 [1.05–1.54], *p* = 0.013). Gender-specific patterns were also observed. Among females, NSSI DSM-5 risk was additionally associated with living with father or mother (vs. parents) (OR = 2.49 [1.11–5.62], *p* = 0.027), recent stressful events (OR = 2.50 [1.10–6.12], *p* = 0.035), and substance use (OR = 3.32 [1.25–8.91], *p* = 0.016). Among males, NSSI DSM-5 risk was uniquely associated with a primary diagnosis of reaction to severe stress and adjustment disorders (OR = 17.2 [2.03–171], *p* = 0.011), in addition to the shared predictors above.

**Table 4:** Logistic regression for NSSI DSM-5 risk, stratified by gender.

	Female				Male			
	Crude		Adjusted		Crude		Adjusted	
Variable	OR (95% CI)	p-value	OR (95% CI)	p-value	OR (95% CI)	p-value	OR (95% CI)	p-value
**Age**	0.94 (0.85 - 1.04)	0.215	0.88 (0.76 - 1.01)	0.066	1.05 (0.88 - 1.26)	0.611	0.77 (0.58 - 1.02)	0.067
Educational level								
< High school	—				—			
High school and above	0.91 (0.59 - 1.43)	0.690			1.57 (0.73 - 3.51)	0.254		
Living area								
Urban	—		—		—		—	
Rural	0.94 (0.60 - 1.46)	0.785	1.03 (0.59 - 1.81)	0.91	0.88 (0.38 - 1.91)	0.755	2.09 (0.71 - 6.20)	0.18
Sexual orientation								
Heterosexual	—		—		—		—	
Non-heterosexual	3.21 (1.69 - 6.09)	**<0.001**	1.99 (0.88 - 4.51)	0.10	1.97 (0.43 - 6.65)	0.315	0.90 (0.11 - 5.81)	0.92
Unknown/Unanswer	1.41 (0.88 - 2.26)	0.155	1.08 (0.59 - 1.99)	0.80	1.03 (0.44 - 2.30)	0.934	0.46 (0.14 - 1.35)	0.17
Income								
≤ 10 million VND	—		—		—		—	
> 10 million VND	1.18 (0.77 - 1.81)	0.436	1.19 (0.69 - 2.07)	0.53	1.49 (0.70 - 3.27)	0.305	2.33 (0.83 - 5.62)	0.23
Living with								
Parents	—		—		—		—	
Father or Mother	2.57 (1.41 - 4.61)	**0.002**	2.49 (1.11 - 5.62)	**0.027**	0.90 (0.21 - 2.74)	0.872	0.35 (0.04 - 1.99)	0.28
Others	1.61 (0.67 - 3.57)	0.259	1.16 (0.39 - 3.32)	0.78	0.68 (0.04 - 3.58)	0.716	0.16 (0.01 - 1.22)	0.12
Parents relationships								
Harmonious	—				—			
Conflicting, lived together	1.27 (0.74 - 2.13)	0.372			2.93 (1.19 - 6.77)	**0.015**		
Divorced/Separated/ Single parent	2.63 (1.43 - 4.79)	**0.002**			0.93 (0.14 - 3.43)	0.923		
Parents-child relationship								
Warmth/secured attachment	—		—		—		—	
Less caring/ communication	2.06 (1.19 - 3.61)	**0.010**	0.60 (0.27 - 1.28)	0.19	2.62 (1.05 - 6.91)	**0.042**	2.51 (0.67 - 10.3)	0.18
Critical manner	4.00 (2.11 - 7.68)	**<0.001**	0.80 (0.33 - 1.87)	0.60	3.95 (0.99 - 13.7)	**0.036**	0.99 (0.12 - 6.92)	>0.99
Others	4.12 (2.05 - 8.31)	**<0.001**	1.70 (0.70 - 4.09)	0.24	3.77 (1.17 - 11.6)	**0.020**	3.69 (0.80 - 17.4)	0.091
Family history of self-harm								
No	—		—		—		—	
Yes	6.97 (4.33 - 11.3)	**<0.001**	4.41 (2.48 - 7.95)	**<0.001**	4.57 (1.83 - 10.7)	**<0.001**	3.15 (1.13 - 10.5)	**0.042**
Recent stressful events								
No	—		—		—		—	
Yes	4.37 (2.47 - 8.28)	**<0.001**	2.50 (1.10 - 6.12)	**0.035**	6.24 (2.64 - 17.2)	**<0.001**	3.18 (0.95 - 11.8)	0.070
CTQ-SF subscales								
Physical abuse	1.19 (1.12 - 1.27)	**<0.001**	1.02 (0.94 - 1.12)	0.61	1.11 (0.99 - 1.22)	0.057	0.94 (0.75 - 1.14)	0.53
Emotional abuse	1.20 (1.15 - 1.26)	**<0.001**	1.09 (1.02 - 1.17)	**0.014**	1.21 (1.11 - 1.32)	**<0.001**	1.26 (1.08 - 1.49)	**0.004**
Sexual abuse	1.09 (0.99 - 1.20)	0.064	0.90 (0.78 - 1.02)	0.11	1.02 (0.73 - 1.22)	0.884	0.85 (0.49 - 1.17)	0.45
Emotional neglect	1.15 (1.09 - 1.20)	**<0.001**	1.06 (0.99 - 1.14)	0.094	1.09 (1.02 - 1.18)	**0.017**	1.05 (0.92 - 1.21)	0.47
**MSI**–**BPD score**	1.33 (1.23 - 1.44)	**<0.001**	1.17 (1.06 - 1.30)	**0.003**	1.38 (1.22 - 1.58)	**<0.001**	1.27 (1.05 - 1.54)	**0.013**
Substance use								
No	—		—		—		—	
Yes	3.58 (1.62 - 7.95)	**0.002**	3.32 (1.25 - 8.91)	**0.016**	0.73 (0.11 - 2.62)	0.681	0.28 (0.03 - 1.48)	0.17
Primary (main) clinical diagnosis								
Depressive disorder								
No	—		—		—		—	
Yes	1.92 (1.09 - 3.33)	**0.021**	0.97 (0.46 - 2.05)	0.95	2.15 (0.60 - 6.19)	0.188	1.60 (0.27 - 7.67)	0.57
Anxiety disorder								
No	—		—		—		—	
Yes	0.55 (0.26 - 1.05)	0.088	1.11 (0.45 - 2.61)	0.82	1.48 (0.34 - 4.63)	0.545	2.93 (0.48 - 15.4)	0.21
Reaction to severe stress, and adjustment disorders								
No	—		—		—		—	
Yes	0.97 (0.40 - 2.12)	0.935	0.60 (0.20 - 1.70)	0.35	20.8 (4.83 - 106)	**<0.001**	17.2 (2.03 - 171)	**0.011**

Crude and adjusted models include “Institution” indicators (fixed effects), “Institution” coefficients are not shown.

CI, Confidence Interval; OR, Odds Ratio.

Bold values indicate statistically significant results.

## 4 Discussion

While there is a substantial body of research on NSSI in Western countries, our understanding of this issue in Asian countries remains very limited. In this study, we present—for the first time in Vietnam—findings on NSSI diagnosed according to the DSM-5-TR proposed criteria, which has been shown to provide more precise estimates (23). Our results focus on identifying the prevalence, clinical characteristics, functions, and correlates of NSSI among adolescents aged 10–19 years who presented at mental health institutions.

### 4.1 Prevalence

Among 804 patients aged 10–19 years, the 12-month prevalence of NSSI DSM-5 was 17.9%, with a higher rate in females (24.8%) than in males (8.7%). The participants in this study were outpatients seeking mental health support, most of whom did not require hospitalization. This finding is similar to a study from Canada, which reported a 20.2% prevalence of NSSI DSM-5 among 2,013 adolescents with mental health needs (24). In contrast, studies conducted in inpatient psychiatric settings have found much higher rates. For example, one study reported a 38.5% prevalence among inpatients with an average age of 15.1 years (25), while another found rates up to 50% (26). Other research has indicated that more than 70% of inpatients engaged in NSSI behaviors in the past year (27; 28). Compared to clinical settings, studies of NSSI in community populations are more common and often report higher prevalence rates. A recent meta-analysis of 264,638 non-clinical adolescents indicated a 12-month prevalence of NSSI at 23.2% (4). However, these studies typically use self-report checklists or questionnaires, which tend to yield higher estimates than clinical diagnosis or single-item questions (29). For instance, among 3,060 adolescents aged 15–17 years, 35.6% reported NSSI during the past year, but only 6.7% fulfilled the NSSI DSM-5 criteria (23). This pattern is also observed in adult populations (30). The prevalence of NSSI DSM-5 peaks during adolescence and young adulthood (ages 14–24) and decreases with age (31). To our knowledge, only one English-language, peer-reviewed study has examined NSSI behaviors using the FASM in Vietnamese adolescents and reported that 43.9% experienced at least one type of NSSI behavior within the preceding 12 months (13). Notably, in our study, 36% of all patients were at high risk of repeating self-harm behaviors, as measured by the ABUSI questionnaire.

### 4.2 Characteristics

We found that the age of onset for NSSI behavior ranges from 12 to 15 years, with an average of 13.50 years in males and 13.11 years in females. Our results are consistent with previous studies conducted in both clinical and non-clinical settings. For example, studies in Western countries have reported an average onset age of 12.52–13.05 years (SD 1.73–3.53) (9), while studies in Southeast Asian countries have found the onset to be between 13 and 16.5 years (32). Overall, these findings indicate that NSSI most frequently begins in early adolescence. However, a major proportion of these studies have recruited participants who are children, adolescents, or young adults. A community-based study in Germany that included participants aged 14–94 years reported an average age of onset for NSSI (DSM-5-based diagnosis) of 17.25 ± 8.9 years (31). Among the 12 common NSSI act presentations reported in our study, scratching and cutting were the most frequently endorsed methods in both genders. Severe forms, such as burning, self bone-breaking, and dropping acid on the skin, were less common. Although both genders shared similar NSSI method patterns to some extent, females were more likely to endorse blood-related methods such as cutting and scratching, while males tended to express their emotional discomfort through aggressive behaviors, such as hitting, more often than females. Our results align with a previous meta-analysis, which found that over 70% of adolescents engaging in NSSI reported self-cutting, often accompanied by scratching, hitting, and burning (2). In a school-based study of NSSI among Vietnamese adolescents, minor behaviors such as intentional self-hitting, wound picking, and self-biting were more prevalent than moderate or severe behaviors, including cutting, carving, scraping, or burning the skin (13). NSSI methods also vary across age groups and study contexts. For example, head banging, carving, hitting, or bruising are more common in young adults (33), while self-biting and self-hitting are prevalent among adolescents and young adults in another report (34). In addition to these different method patterns, females who engaged in NSSI also seemed to employ more NSSI methods than males. Regarding the anatomical locations of NSSI acts, females tended to perform injuries on more areas of the body than their male counterparts. Our results did not indicate significant differences between genders overall, except that females were more likely to self-injure their arms and forearms compared to males. In contrast, male patients tended to engage in acts on the face more than females, although this difference approached statistical significance. These findings are consistent with previous studies, which found that female adolescents were more likely than males to injure their arms and legs, whereas males were more likely to self-injure their face and chest (35, 36).

To date, explanations for gender disparities in NSSI presentations remain limited (2). Psychologically, the choice of NSSI methods partly reflect differences in emotional expression and coping styles. Adolescent females are more likely to internalize emotional distress, leading to self-directed behaviors such as cutting or scratching, often to relieve internal pain or emotional numbness and regain a sense of control (37, 38). In contrast, males tend to externalize distress through outwardly aggressive behaviors, such as hitting objects or themselves, to express anger or frustration (37). These patterns are influenced by social acceptability and gender norms: males are expected to express emotions outwardly, making visible injuries (such as on the face, as observed in this study) more socially acceptable or ignored, whereas females often injure more easily concealed areas (e.g., arms, forearms), possibly due to concerns about body image, stigma, or a desire to hide wounds (35, 39). Recent research has increasingly focused on neurobiological aspects of NSSI, especially pain-related characteristics. Studies have shown that individuals with NSSI exhibit higher pain thresholds, pain tolerance, total pain index, and pain experience intensity compared to healthy controls (40). Functional MRI studies suggest that pain inhibition was enhanced and activation of pain modulatory brain networks was increased in NSSI (41). However, gender differences in neural responses to physical pain and their impact on the regulation of emotional pain—and thus on the choice of NSSI methods—remain largely unknown. These findings are primarily drawn from studies conducted in Western countries. We suppose that the similarities in NSSI clinical manifestations, to some extent, may be relevant in explaining the results of our study.

The majority of NSSI acts occurred when NSSI DSM-5 participants were alone, with no significant gender differences in the environmental situation during the act. This finding suggests that NSSI behaviors are primarily solitary acts, regardless of gender. This pattern may reflect feelings of shame, secrecy, or a desire to avoid detection and intervention by others, rather than an attempt to seek attention or attract others. Understanding the functions of NSSI acts provides clinicians and researchers with greater insight into the characteristics of this condition.

### 4.3 Functions

From the results of the current study, adolescents engaged in NSSI behaviors primarily to alleviate internal emotional distress. There were no significant gender differences in the purposes of NSSI, which most commonly involved automatic negative reinforcement (e.g., “to stop bad feelings”, “to deal with anger”, “to deal with disappointment”) and automatic positive reinforcement (e.g., “to feel relaxed”, “to punish self”). Participants also reported social reinforcement motives, such as “to get care from parents or friends” or “to avoid school, work, or any social interaction”, though these were less frequent. Our findings among adolescents seeking mental health support are consistent with previous studies across diverse populations and contexts (2, 7, 42). In addition to the characteristics of NSSI in terms of form and context, it is evident that NSSI is used as a coping mechanism for internal conflicts and uncomfortable feelings. Research utilizing ecological momentary assessment has found that individuals often experience heightened loneliness, sadness, and feelings of being overwhelmed prior to engaging in NSSI (43). Results from a meta-analysis examining NSSI and affect regulation indicated that experiencing pain is associated with reductions in negative emotions (44). Notably, individuals engaging in NSSI often have multiple overlapping motives, with the most common combination involving both automatic negative and positive reinforcement. These individuals typically seek relief and relaxation after NSSI to deal with negative mood states, highlighting both emotional dysregulation and a lack of effective coping strategies. Over time, this maladaptive cycle can exacerbate the severity of the condition. When NSSI strategies lose their effectiveness (desensitization), there is an increased risk of suicidal behaviors (45). Psychotherapies focusing on emotion regulation and coping skills for adverse situations—such as cognitive behavioral therapy, dialectical behavior therapy, and emotion-regulation group therapy—may provide benefit. Based on the primary motives for NSSI, the selection of psychological intervention strategies should be tailored to the individual needs of each patient.

### 4.4 Correlates

The potential risk factors for NSSI can be categorized into environmental and individual factors. In our study, family history of self-harm, greater childhood emotional abuse, and higher MSI–BPD scores were significantly associated with increased NSSI DSM-5 risk in both genders.

The term ‘self-harm’ in our family history measure encompasses both nonsuicidal self-injury and suicidal self-harm behaviors; therefore, it is broader than a family history of NSSI specifically. Because this finding is based on observational data and our measure does not distinguish suicidal from non-suicidal behaviors in relatives, the association should be interpreted as familial clustering rather than direct evidence of NSSI heritability. The observed relationship may reflect genetic factors, shared environmental influences, or both, contributing to the intergenerational transmission of self-injurious behaviors. Evidence from twin and family studies suggests that NSSI is moderately heritable, with genetic factors accounting for a substantial proportion of the variance—estimated at 37% for men and 59% for women (46, 47). Additionally, genome-wide association studies have reported NSSI heritability to be approximately 10% (48). Beyond genetic influences, elevated risk of NSSI may also result from social learning and modeling processes, through which maladaptive behaviors are acquired within dysfunctional family environments (49). Family history thus serves as a potent model for behavior, and families with a history of self-harm may transmit maladaptive emotional regulation strategies, further increasing vulnerability to NSSI in offspring (49, 50).

Experiencing adverse childhood experiences (ACEs)—emotional abuse specifically in this study—is positively correlated with risk of NSSI DSM-5. ACEs, especially early-life maltreatment, are among the strongest risk factors not only for NSSI, but also for other psychiatric and mental disorders later in life (51–53). In Vietnam, emotional abuse has been reported as the most common form of ACE, affecting approximately 21.9–42.3% of participants across studies (54, 55). Repeated experiences of abuse during childhood or adolescence, from a psychological perspective, put individuals at risk of isolation, loneliness, and a lack of effective communication and sharing with those around them (56). This, both directly and indirectly, reduces the ability to learn necessary developmental skills, including how to manage stressful events and regulate emotions effectively (52). Educational approaches aimed at preventing ACEs are generally considered appropriate strategies for reducing health risks, including NSSI.

A higher MSI–BPD score was another strong factor associated with NSSI DSM-5 in our study. We assessed the propensity for BPD using a questionnaire rather than clinical diagnosis, due to the need for diagnostic caution in individuals under 18 years. This association is theoretically expected, as the link between NSSI and BPD has been long recognized. Previous research shows that a high proportion of inpatients with NSSI meet criteria for BPD (57), and nearly all adolescents with BPD report a history of NSSI, with an average of almost five different types of self-injury. Moreover, greater BPD severity in adolescents is associated with increased engagement in NSSI (58). However, NSSI can occur both alongside and independently of BPD traits (59), and studies emphasize that NSSI is not necessarily indicative of BPD; the diagnosis of NSSI DSM-5 may simply coincide with BPD (26). While our data were insufficient to support an independent diagnosis of NSSI disorder apart from BPD, screening for BPD traits in clinical settings may be useful for guiding targeted interventions.

In our study cohort, “experiencing recent stressful events” was identified as a risk factor for NSSI DSM-5 in females, while “reaction to severe stress and adjustment disorders” was identified as a risk factor for NSSI DSM-5 in males. This finding is consistent with previous research, including a meta-analysis of 21 studies that reported a significant association between daily stressful events and NSSI, with an adjusted pooled OR of 1.33 (95% CI = 1.08–1.63) (60). This meta-analysis also found that the association between life stress and NSSI was similar across different types of stressors—whether traumatic, general, or family-related—indicating that various categories of stress have comparable impacts on NSSI risk. Life stress is recognized as a non-specific risk factor not only for NSSI but also for psychiatric disorders more broadly. Its influence on NSSI is thought to be mediated by psychological and emotional mechanisms, such as depression (61), anxiety (62), difficulty in emotion regulation, and internalizing symptoms (63). Besides, living with single parent, and history of substance use were also showed significant association with higher risk of NSSI DSM-5 in female. Disruptions in the family environment are considered chronic stressors and have been widely identified as risk factors for adolescent maladaptive behaviors, including NSSI. (64, 65). Substance use may represent either a risk factor for, or consequence of, the emotional dysregulation commonly observed in NSSI, potentially creating a cycle of maladaptive coping behaviors (51). These findings suggest a “cumulative risk” model where environmental stressors and behavioral comorbidities may intersect to heighten NSSI vulnerability. Education focused on managing emotional and cognitive responses during stressful periods may be an effective strategy to reduce the risk of maladaptive behaviors in both genders.

In this study, we observed a significantly higher prevalence of females meeting NSSI DSM-5 criteria than males among patients seeking mental health support. However in Vietnam, which is characterized by a collectivist culture, societal norms may discourage males from expressing vulnerability or emotional distress, in contrast to more individualistic Western societies. This may potentially cause Vietnamese males to be less likely to seek professional support or disclose emotional crises. Evidence from other contexts suggests that subgroup differences in clinical presentations may be strongly influenced by disruptions in access to care and case detection. For example, reduced psychiatric admissions among older adults were reported during the COVID-19 lockdown, with proposed mechanisms including hospital avoidance due to fear of contagion and reduced opportunities to identify behavioral changes (66). In Western and high-income countries, female sex is consistently associated with a higher risk of NSSI (51, 67). However, recent systematic reviews have found no significant gender difference in NSSI risk in Asian countries (68), including Southeast Asia (7), and in some South Asian countries, males are even at greater risk than females (8). Taken together with these mixed findings on gender differences in NSSI across Asian settings, our results raise the possibility that NSSI in Vietnamese males may be under-detected or under-disclosed in service settings, reinforcing the need to consider culturally patterned symptom expression and pathways to care when interpreting diagnostic prevalence. These findings highlight the influence of cultural heterogeneity on the presentation of NSSI and raise concerns about the adequacy of NSSI diagnosis in males, who may exhibit different patterns of help-seeking or symptom expression, particularly in non-Western contexts.

### 4.5 Strengths and limitations

Our study offers several important strengths that enhance the credibility of its findings. We focused on individuals who met established clinical criteria for NSSI as determined by senior psychiatrists. This clinical evaluation approach, rather than reliance on rating scales alone, allowed for a more accurate and clinically meaningful assessment. Nevertheless, some limitations regarding (1) study design, sampling and (2) statistical analysis should be considered when interpreting our results:

- Regarding the study design and sampling, researchers in our study were comprehensively trained to diagnose NSSI using a semi-structured questionnaire developed based on DSM-5-TR and piloted on a small sample, which could minimize diagnostic variance between raters to some extent. However, the lack of inter-rater reliability measurement on blinded samples due to contextual constraints represents a limitation that could affect the reliability of our results. During recruitment, 10 participants refused participation without providing specific reasons, which might introduce selection bias in our prevalence estimates if they had undisclosed self-harm behaviors. Since participants were drawn exclusively from the general psychiatry departments, our sample did not include individuals from psychiatric departments of child and adolescent hospitals, which may limit the generalizability of our findings. There is also the possibility that males were underrepresented in our study, potentially due to lower mental health awareness or a reduced likelihood of seeking help, which could have influenced gender-related observations. Another issue in our study is that mental disorders were diagnosed using ICD-10 which is the official diagnostic system in Vietnam, whereas NSSI was defined using DSM-5 criteria. Although these diagnostic systems overlap, they are not fully equivalent in their definitions and thresholds, which may introduce classification inconsistency and complicate interpretation of associations (66). This mismatch could have led to non-differential misclassification and may have biased effect estimates toward the null. Additionally, the reliance on retrospective self-reports introduces the risk of recall bias, particularly among participants experiencing emotional disturbances, who may be prone to over-reporting symptoms. The presence of comorbid psychiatric conditions further complicates the interpretation of the relationship between mental disorders and NSSI DSM-5, and may differ from patterns observed in community-based samples. The clinical setting imposed certain constraints, such as limited time for assessment and reduced capacity for in-depth exploration, resulting in a predominance of quantitative data over qualitative insights.
- Regarding statistical analysis, our study included only three institutions, so we adjusted for institution using fixed effects; however, with only three clusters, inference that accounts for within-institution correlation is limited, making standard errors and p-values potentially unreliable. Additionally, given the small number of males with NSSI DSM-5, gender comparisons may be underpowered, especially after multiple testing corrections. Our exploratory analysis of gender differences should be considered preliminary and suggestive of directions for larger studies. Specifically, results that did not survive multiple testing corrections should be interpreted with particular caution.

These factors collectively suggest that while our findings provide valuable clinical insights, they should be interpreted with an awareness of these methodological considerations.

## 5 Conclusions

In conclusion, this study identified a high prevalence of Vietnamese adolescents seeking mental health services who met the DSM-5-TR criteria for NSSI. These findings are important in raising awareness among Vietnamese psychiatrists about NSSI as an independent mental disorder, which is not yet recognized in the ICD-10 classification. By providing initial data on the clinical diagnosis of NSSI, our study provides valuable insights into this issue within the context of a low- and middle-income country with unique cultural characteristics. Further research is warranted to develop effective strategies for the diagnosis, treatment, and management of NSSI in this population.

## Data Availability

The raw data supporting the conclusions of this article will be made available by the authors, without undue reservation.
