# Supplementary material for: Assessing the prevalence, characteristics and psychosocial correlates of nonsuicidal self-injury among Vietnamese adolescent psychiatric outpatients: a cross-sectional study
Source: Front Psychiatry. 2026 Feb 18;17:1699844. doi: 10.3389/fpsyt.2026.1699844 (PMC12957150; doi:10.3389/fpsyt.2026.1699844)
Supplement: Supplementary file 1 [file Table1.docx]

*Supplementary material 1:*

Algorithm to diagnose NSSI DSM-5:

**Start from the Q1 to Q6 (for Criterion A).**

- If Criterion A is met, continue the rest of the questionnaire.
- If Criterion A is not met, stop the NSSI DSM-5 investigation.

Q1. When you're stressed or in negative emotional state, what do you usually do to feel better?

Q2. Have there been times you intentionally hurt your body on purpose?

| **Criteria**  **(Main points)** | **Open-ended core questions** | **Probes**  **(where applicable)** |
| --- | --- | --- |
| **A.**   - **Past-year frequency** - **Intentionality** - **Expected severity** - ***No suicidal intent*** | Q3. If there were times you intentionally hurt your body on purpose, how about those behaviors over the last 12 months? Can you describe in details? | - What happened? - What did you do to your body? - Where on the body? - What usually led up to it? |
|  | Q4. When you did that, what were you hoping would happen physically? | - Were you expecting bleeding, bruising, pain, or something else? - Were you trying to cause serious injury, or more minor/moderate harm? |
|  | Q5. At the time, what did you want to happen to you overall? | - Were you trying to end your life, or was it for another reason? |
|  | Q6. Walk me through the last year, how many separate days did this happen? | - Was it clustered into a few weeks or spread out? - What’s your best estimate of the number of days? - When was the most recent time? |

Confirm:

A1. In the **past 12 months**, you intentionally caused damage to the surface of my body likely to cause pain, bruising, or bleeding (examples: cutting, burning, stabbing, hitting, excessive rubbing). (☐ Yes ☐ No ☐ Not sure)

A2. This occurred on **5 or more separate days** in the past 12 months. (☐ Yes ☐ No ☐ Not sure)

A3. At the time, you expected the injury would cause only **minor or moderate physical harm** (not likely to be lethal). (☐ Yes ☐ No ☐ Not sure)

A4. At the time, you had **no intent to die** from the act(s). (☐ Yes ☐ No ☐ Not sure)

**Criterion A:** ☐ Met ☐ Not met ☐ Not clear

| **Criteria**  **(Main points)** | **Open-ended core questions** | **Probes**  **(where applicable)** |
| --- | --- | --- |
| **B. Expectations/ Functions of the behaviors**   - **Relief** - **Interpersonal** - **Positive feeling** | Q7. What were you hoping the self-injury would do for you in that moment? | - What feeling were you trying to get away from? - Were you trying to feel something positive: calm, relief, control, real, grounded? - Was it meant to solve a situation with someone else? |
|  | Q8. What changed for you during or shortly after you hurt yourself? | - Emotions?” Thoughts? Body sensations? - How long did the change last? |
|  | Q9. Did you ever feel like you needed to do it again to get the same relief or effect? | - Did urges increase over time? - Did it become a go-to response? |

Confirm

In the past 12 months, when you self-injured, did you do it with the expectation that it would…” (check all that apply)

B1. …**relieve a negative feeling or cognitive state** (e.g., anxiety, numbness, shame, self-criticism). ☐ Yes ☐ No ☐ Not sure

B2. …**resolve an interpersonal difficulty** (e.g., reduce conflict, communicate distress, influence closeness/distance). ☐ Yes ☐ No ☐ Not sure

B3. …**induce a positive feeling state** (e.g., calm, relief, feeling real, control). ☐ Yes ☐ No ☐ Not sure

The desired relief/response was experienced **during or shortly after** self-injury. ☐ Yes ☐ No ☐ Not sure

The patterns of behaviors suggesting dependence ☐ Yes ☐ No ☐ Not sure

**Criterion B:** ☐ Met ☐ Not met ☐ Not clear (Met if ≥1 of B1 - B3 = Yes)

| **Criteria**  **(Main points)** | **Open-ended core questions** | **Probes**  **(where applicable)** |
| --- | --- | --- |
| **C. Associated features:**   - **Antecedent distress** - **Preoccupation/urge** - **Frequent thoughts** | Q10. In the hours or day before you self-injured, what was going on emotionally and mentally? | - Depression, anxiety, tension, anger, distress, self-criticism? - Any specific trigger events? |
|  | Q11. Before you did it, did you find yourself fixated on the idea like you couldn’t get it out of your mind? | - How long did that last? - How hard was it to resist? - What helped you resist (if anything)? |
|  | Q12. How often do thoughts about self-injury show up when you don’t act on them? | - Daily? weekly? only during stress? - Do they feel intrusive or controllable? |

Confirm

C1. Immediately prior to self-injury, you often experienced **interpersonal difficulty** or **negative feelings/thoughts** (e.g., depression, anxiety, tension, anger, generalized distress, self-criticism). ☐ Yes ☐ No ☐ Not sure

C2. Prior to self-injury, you experienced a **preoccupation/urge** that was **difficult to control**. (☐ Yes ☐ No ☐ Not sure)

C3. You **frequently think** about self-injury even when you do not act on it. (☐ Yes ☐ No ☐ Not sure)

**Criterion C:** ☐ Met ☐ Not met ☐ Not clear (Met if ≥1 of C1 - C3 = Yes.)

| **Criteria**  **(Main points)** | **Open-ended core questions** | **Probes**  **(where applicable)** |
| --- | --- | --- |
| **D.**   - **Not socially sanctioned** - **Not limited to scab picking/nail biting** | Q13. In your educational, cultural, or religious background, are those behaviors generally considered acceptable by others? | - Are those behaviors unrelated to art or to your cultural or religious background? |
|  | (In case patients presents minor grooming habits like scab picking/nail biting, etc) Q14. When these injuries occur, would you describe them as accidental, part of a habit, or intentional? | - How is this different from things like nail biting, skin picking, or scratching without intending injury? |

**Criterion D:** ☐ Met ☐ Not met ☐ Not clear

| **Criteria**  **(Main points)** | **Open-ended core questions** | **Probes**  **(where applicable)** |
| --- | --- | --- |
| **E. Clinically significant distress or interference (functional impact)** | Q15. How have those behaviors affected your life? | - Interpersonal: “Relationships, trust, conflict, isolation, hiding injuries? - Academic/Work: “Missed school/work, concentration, performance? - Daily functioning**:** “Sleep, routines, self-care, medical needs? - Emotional impact: “Shame, fear, feeling out of control, distress about doing it? - Others? |
|  | Q16. What have been the consequences for you: physically, emotionally, socially? | - Medical attention needed? - Scars you worry about? - Avoiding activities due to concealment? - Others? |
|  | Q17. How distressing is it for you that this happens? | - How distressing is it for you that this happens? |

Confirm:
E1. The behavior and/or its consequences caused **clinically significant distress** (e.g., shame, worry, feeling unable to stop). (☐ Yes ☐ No ☐ Not sure)

E2. The behavior and/or its consequences caused **interference** in important areas of functioning (interpersonal, academic/work, other). (☐ Yes ☐ No ☐ Not sure)

- If Yes: domains impacted (check all): ☐ interpersonal ☐ school/work ☐ family ☐ health ☐ other: ___

**Criterion E:** ☐ Met ☐ Not met ☐ Not clear) (Met if E1 or/and E2 = Yes).

| **Criteria**  **(Main points)** | **Open-ended core question** |
| --- | --- |
| **F. Exclusion / Differential module** | These questions check whether any self-injury happened only during periods of intoxication/withdrawal, severe confusion, or unusual experiences like hearing voices.  Q18. During any self-injury episode, were you experiencing anything unusual like hearing/seeing things others couldn’t, feeling extremely paranoid, or having beliefs others said weren’t true?  Q19. During any self-injury episode, were you ever acutely confused or disoriented - like from illness, high fever, injury, or not knowing where you were?  Q20. Did any self-injury happen when you were intoxicated or high, or when you were withdrawing or coming down?  Q21. Does the self-injury ever happen in a repetitive, automatic way (like head banging/biting/hitting) without a clear goal like relief - more like a habit or repetitive movement? |

**Criteria F: Questions + clinical examination:** ☐ Met ☐ Not met ☐ Not clear (Met if no suspected issues could cause the behaviors)
