# Supplementary material for: Assessing the prevalence, characteristics and psychosocial correlates of nonsuicidal self-injury among Vietnamese adolescent psychiatric outpatients: a cross-sectional study
Source: Front Psychiatry. 2026 Feb 18;17:1699844. doi: 10.3389/fpsyt.2026.1699844 (PMC12957150; doi:10.3389/fpsyt.2026.1699844)
Supplement: Supplementary file 2 [file Table2.docx]

*Supplementary material 2:*

**Nonsuicidal Self-Injury (NSSI) functions validation**

**1. The Content Validity Index (CVI):**

- Item-level CVI (I-CVI) = 1
- Scale-level CVI (S-SVI) = 1

**2. Confirmatory factor analysis**

Using the FASM questionnaire as a primary source for item content, we operationalized NSSI reason items. NSSI reasons were administered and coded as binary (Yes/No) endorsements. Item endorsement patterns were examined prior to factor analysis; some functions showed no endorsement (zero variance), leaving 13 items with non-zero variance. Items with zero variance were not eligible for covariance-based factor modeling and were therefore excluded from confirmatory factor analysis (CFA).

NSSI functions are commonly conceptualized within four functional domains: Automatic Negative Reinforcement (AN), Automatic Positive Reinforcement (AP), Social Negative Reinforcement (SN), and Social Positive Reinforcement (SP) (Nock and Prinstein 2004). Within the endorsed items, only one item (item f5) mapped theoretically to the SN domain. Because a latent factor with a single indicator is not identified in standard CFA without strong constraints, SN was not modeled as a latent variable.

A categorical CFA was therefore conducted on the remaining 12 dichotomous items, specifying a three-factor model (AN, AP, SP) consistent with theory and using an Weighted least squares mean and variance adjusted (WLSMV) estimator. The SN item was retained as an observed binary variable representing for SN.

**
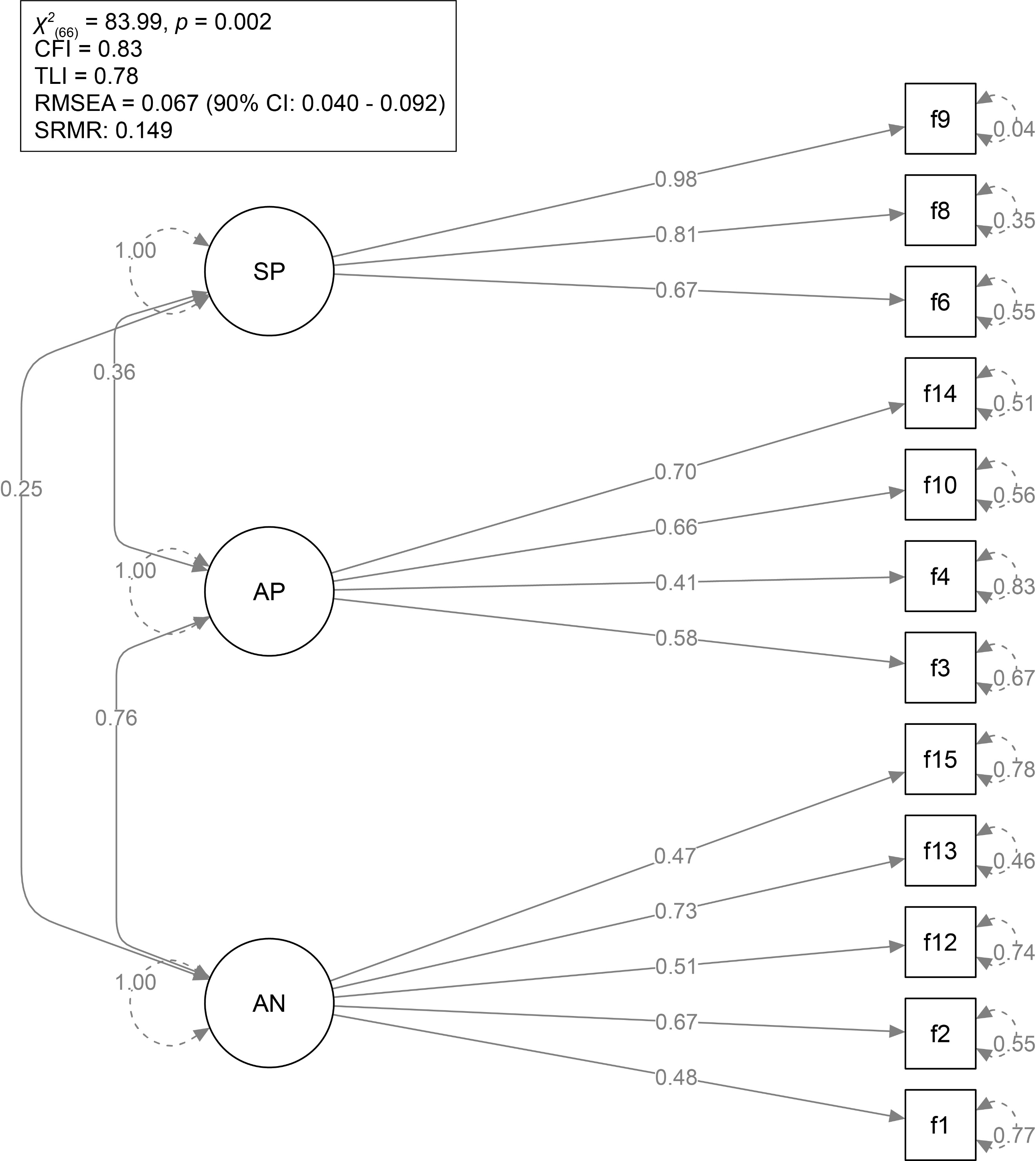
**

**Figure S2-1**. **Confirmatory factor analysis (CFA) of NSSI function items**. A three-factor correlated model was estimated for Automatic Negative Reinforcement (AN), Automatic Positive Reinforcement (AP), and Social Positive Reinforcement (SP); the Social Negative Reinforcement (SN) factor was not modeled because only one indicator was available (item f5). Model fit was evaluated using robust (WLSMV-scaled) fit indices. Overall, results indicated mixed model fit: CFI/TLI and RMSEA suggested acceptable fit, whereas the residual-based index (SRMR) suggested notable misfit. Standardized factor loadings were moderate to strong overall (range 0.41–0.98) and statistically significant (*p* < 0.001), supporting the intended three-factor structure.

**Measurement invariance analysis:** Configural, metric, and scalar invariance across age groups were tested, and practical changes were evaluated primarily using changes in fit indices (ΔCFI, ΔTLI, ΔRMSEA, ΔSRMR). For models estimated with Weighted least squares mean and variance adjusted (WLSMV) method, scaled CFI, TLI, RMSEA and SRMR were used.

*** Age groups were “< 15 years” and “≥ 15 years” (Tables S2-1 and S2-2).**

**Table S2-1.** Measurement invariance across age: Model fit indices (configural, metric, scalar)

| **Model** | **Constraints** | **df** | **χ²*** | **CFI** | **TLI** | **RMSEA** | **SRMR** |
| --- | --- | --- | --- | --- | --- | --- | --- |
| Configural | Same factor structure across age | 102 | 126.45 | 0.847 | 0.802 | 0.073 | 0.171 |
| Metric | + equal factor loadings | 111 | 136.7 | 0.873 | 0.849 | 0.064 | 0.176 |
| Scalar | + equal loadings + equal thresholds | 120 | 145.6 | 0.868 | 0.855 | 0.063 | 0.176 |

** The “χ²” column contains standard test statistics.*

**Table S2-2**. Measurement invariance across age: scaled χ² difference tests and changes in fit indices (ΔCFI, ΔTLI, ΔRMSEA, ΔSRMR)

| **Comparison** | **Δdf** | **Δ χ²** | ***p*** | **ΔCFI** | **ΔTLI** | **ΔRMSEA** | **ΔSRMR** |
| --- | --- | --- | --- | --- | --- | --- | --- |
| Metric vs. Configural | 9 | 6.81 | 0.65 | 0.016 | 0.047 | -0.009 | 0.005 |
| Scalar vs. Metric | 9 | 11.53 | 0.24 | -0.007 | 0.006 | -0.001 | 0.000 |

The scaled χ² difference tests indicated no statistically significant worsening of fit when moving from the configural model to the metric model (Δχ² = 6.81, Δdf = 9, *p* = 0.65) or from the metric model to the scalar model (Δχ² = 11.53, Δdf = 9, *p* = 0.24). Changes in practical fit indices were small across steps (configural to metric: ΔCFI = 0.016, ΔTLI = 0.047, ΔRMSEA = -0.009, ΔSRMR = 0.005; metric to scalar: ΔCFI = −0.007, ΔTLI = 0.006, ΔRMSEA = −0.001, ΔSRMR = 0.000), supporting overall measurement invariance across age under the tested constraints.

Measurement invariance across gender was not evaluated because the male subgroup was small (n = 30) and several items showed empty cells (no observed “Yes” endorsements), which prevented stable estimation and model identification for multi-group invariance testing.
