# Supplementary material for: Assessing the prevalence, characteristics and psychosocial correlates of nonsuicidal self-injury among Vietnamese adolescent psychiatric outpatients: a cross-sectional study
Source: Front Psychiatry. 2026 Feb 18;17:1699844. doi: 10.3389/fpsyt.2026.1699844 (PMC12957150; doi:10.3389/fpsyt.2026.1699844)
Supplement: Supplementary file 3 [file Table3.docx]

*Supplementary material 3:*

**The Childhood Trauma Questionnaire – Short Form (CTQ–SF) Scale validation**

**1. The Content Validity Index (CVI):**

- Item-level CVI (I-CVI) = 1
- Scale-level CVI (S-SVI) = 1

**2. Item-item correlation of the CTQ–SF scale**


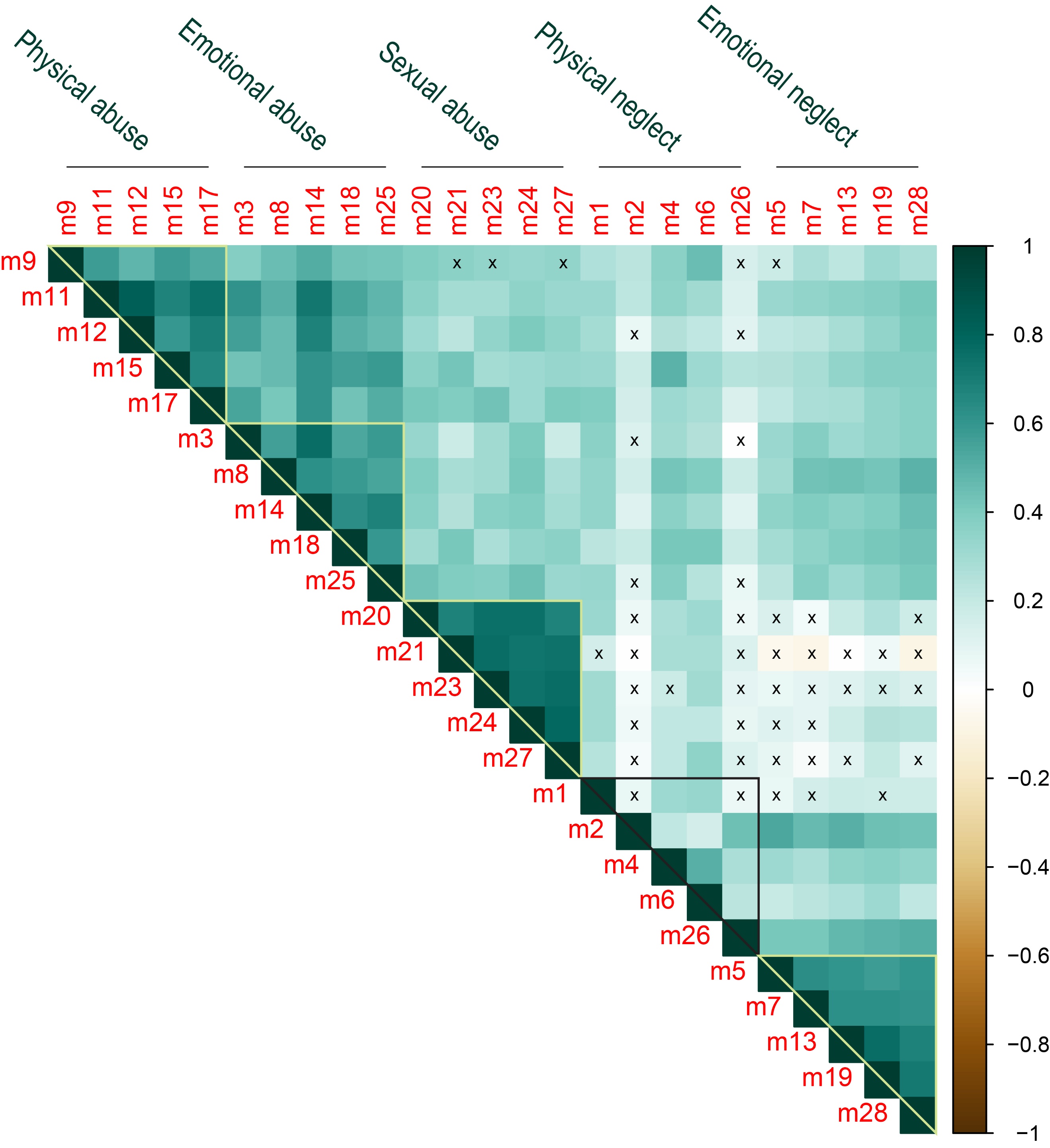


**Figure S3-1**. **Item–item polychoric correlation heatmap for the CTQ–SF, with items grouped by subscale**. Correlations were computed as polychoric correlations, which estimate the association between latent continuous variables underlying the observed binary item responses. Cells marked “x” indicate non-significant correlations (p > 0.05). The physical neglect items exhibit weaker and less consistent inter-item correlations relative to other subscales, suggesting suboptimal psychometric performance; these items were therefore flagged for further evaluation and possible removal in the Vietnamese validated version (see reliability and factor-analytic results).

**3. Confirmatory factor analysis**


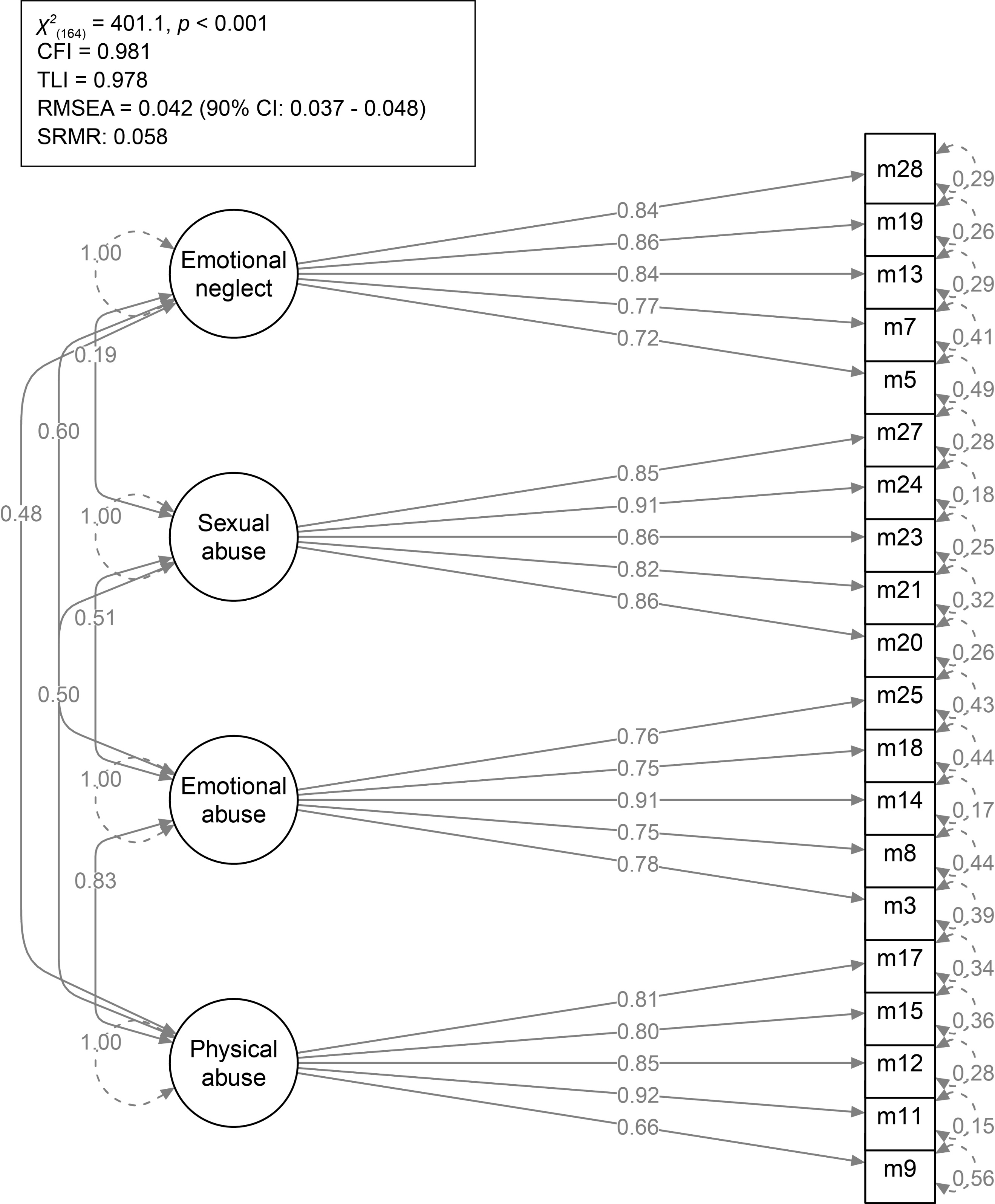


**Figure S3-2**. **Confirmatory factor analysis (CFA) of the CTQ–SF**. A four-factor correlated model (emotional neglect, sexual abuse, emotional abuse, and physical abuse) was fitted after excluding the physical neglect subscale because the initial five-factor model showed poorer model fit. The four-factor model demonstrated good overall fit, with moderate to strong standardized factor loadings (0.66–0.92) and all loadings statistically significant (*p* < 0.001), supporting the revised factor structure. CFI: Comparative Fit Index, TLI: Tucker–Lewis Index, RMSEA: Root Mean Square Error of Approximation, SRMR: Standardized Root Mean Square Residual.

**Measurement invariance analysis:** Configural, metric, and scalar invariance across groups (gender and age) were tested, and practical changes were evaluated primarily using changes in fit indices (ΔCFI, ΔTLI, ΔRMSEA, ΔSRMR). For models estimated with Weighted least squares mean and variance adjusted (WLSMV) method, scaled CFI, TLI, RMSEA and SRMR were used.

*** Gender groups were male and female (Tables S3-1 and S3-2).**

**Table S3-1.** Measurement invariance across gender: Model fit indices (configural, metric, scalar)

| **Model** | **Constraints** | **df** | **χ²*** | **CFI** | **TLI** | **RMSEA** | **SRMR** |
| --- | --- | --- | --- | --- | --- | --- | --- |
| Configural | Same factor structure across age | 328 | 407.05 | 0.983 | 0.981 | 0.039 | 0.067 |
| Metric | + equal factor loadings | 344 | 455.44 | 0.985 | 0.983 | 0.037 | 0.072 |
| Scalar | + equal loadings + equal thresholds | 379 | 440.93 | 0.983 | 0.983 | 0.037 | 0.067 |

** The “χ²” column contains standard test statistics.*

**Table S3-2**. Measurement invariance across gender: Scaled χ² difference tests and changes in fit indices (ΔCFI, ΔTLI, ΔRMSEA, ΔSRMR)

| **Comparison** | **Δdf** | **Δ χ²** | ***p*** | **ΔCFI** | **ΔTLI** | **ΔRMSEA** | **ΔSRMR** |
| --- | --- | --- | --- | --- | --- | --- | --- |
| Metric vs. Configural | 16 | 23.54 | 0.10 | 0.002 | 0.002 | 0.002 | 0.005 |
| Scalar vs. Metric | 35 | -32.04 | 1 | 0.002 | 0.000 | 0.000 | -0.005 |

The scaled χ² difference tests supported invariance across gender groups, indicating no statistically significant decrement in model fit when moving from the configural to the metric model (Δχ² = 23.54, Δdf = 16, *p* = 0.1) or from the metric to the scalar model (Δχ² = -32.04, Δdf = 35, *p* = 1.0). Measurement invariance was also evaluated using changes in practical fit indices. These changes were small from the configural to the metric model (ΔCFI = 0.002; ΔTLI = 0.002; ΔRMSEA = -0.002; ΔSRMR = 0.005) and from the metric to the scalar model (ΔCFI = -0.002; ΔTLI = 0.000; ΔRMSEA = 0.000; ΔSRMR = -0.005), supporting overall measurement invariance across gender.

*** Age groups were “< 15 years” and “≥ 15 years” (Tables S3-3 and S3-4).**

**Table S3-3.** Measurement invariance across age: Model fit indices (configural, metric, scalar)

| **Model** | **Constraints** | **df** | **χ²*** | **CFI** | **TLI** | **RMSEA** | **SRMR** |
| --- | --- | --- | --- | --- | --- | --- | --- |
| Configural | Same factor structure across age | 328 | 386.02 | 0.986 | 0.984 | 0.037 | 0.069 |
| Metric | + equal factor loadings | 344 | 427.11 | 0.988 | 0.987 | 0.034 | 0.073 |
| Scalar | + equal loadings + equal thresholds | 374 | 414.67 | 0.986 | 0.986 | 0.035 | 0.069 |

** The “χ²” column contains standard test statistics.*

**Table S3-4**. Measurement invariance across age: scaled χ² difference tests and changes in fit indices (ΔCFI, ΔTLI, ΔRMSEA, ΔSRMR)

| **Comparison** | **Δdf** | **Δ χ²** | ***p*** | **ΔCFI** | **ΔTLI** | **ΔRMSEA** | **ΔSRMR** |
| --- | --- | --- | --- | --- | --- | --- | --- |
| Metric vs. Configural | 16 | 18.918 | 0.27 | 0.002 | 0.003 | -0.003 | 0.004 |
| Scalar vs. Metric | 30 | -27.154 | 1 | -0.002 | -0.001 | 0.001 | -0.004 |

The scaled χ² difference tests supported invariance across age groups, indicating no statistically significant decrement in model fit when moving from the configural to the metric model (Δχ² = 18.918, Δdf = 16, *p* = 0.27) or from the metric to the scalar model (Δχ² = -27.154, Δdf = 30, *p* = 1.0). Measurement invariance was also evaluated using changes in practical fit indices. These changes were small from the configural to the metric model (ΔCFI = 0.002; ΔTLI = 0.003; ΔRMSEA = -0.003; ΔSRMR = 0.004) and from the metric to the scalar model (ΔCFI = -0.002; ΔTLI = -0.001; ΔRMSEA = 0.001; ΔSRMR = -0.004), supporting overall measurement invariance across age.

**4. Internal inter-item consistence analysis:**

| **CTQ–SF** | **Cronbach’s α** | | **Decision** |
| --- | --- | --- | --- |
| Physical abuse | 0.81 | Good |  |
| Emotional abuse | 0.83 | Good |  |
| Sexual abuse | 0.81 | Good |  |
| Physical neglect | 0.47 | Very poor | Excluded |
| Emotional neglect | 0.87 | Good |  |

Note: In our Vietnamese adolescent sample, the CTQ–SF physical neglect subscale showed poor internal consistency (α = 0.47) and weaker inter-item correlations relative to other subscales, suggesting suboptimal measurement performance in this context. This may reflect cross-cultural differences in how physical neglect is understood and reported, including normative variation in caregiving practices, supervision, and adolescents’ household responsibilities, as well as challenges in adolescents’ recall of early-life material deprivation. In addition, some items may have limited semantic equivalence after translation, leading to heterogeneous interpretation. Accordingly, we excluded the physical neglect subscale from the revised factor model and recommend further validation work (e.g., cognitive interviewing and item refinement) to strengthen assessment of physical neglect in Vietnamese youth.
