# Supplementary material for: Assessing the prevalence, characteristics and psychosocial correlates of nonsuicidal self-injury among Vietnamese adolescent psychiatric outpatients: a cross-sectional study
Source: Front Psychiatry. 2026 Feb 18;17:1699844. doi: 10.3389/fpsyt.2026.1699844 (PMC12957150; doi:10.3389/fpsyt.2026.1699844)
Supplement: Supplementary file 4 [file Table4.docx]

*Supplementary material 4:*

**The McLean Screening Instrument for Borderline Personality Disorder (MSI**–**BPD) validation**

**1. The Content Validity Index (CVI):**

- Item-level CVI (I-CVI) = 1
- Scale-level CVI (S-SVI) = 1

**2. Item-item correlation of the MSI-BPD**

**
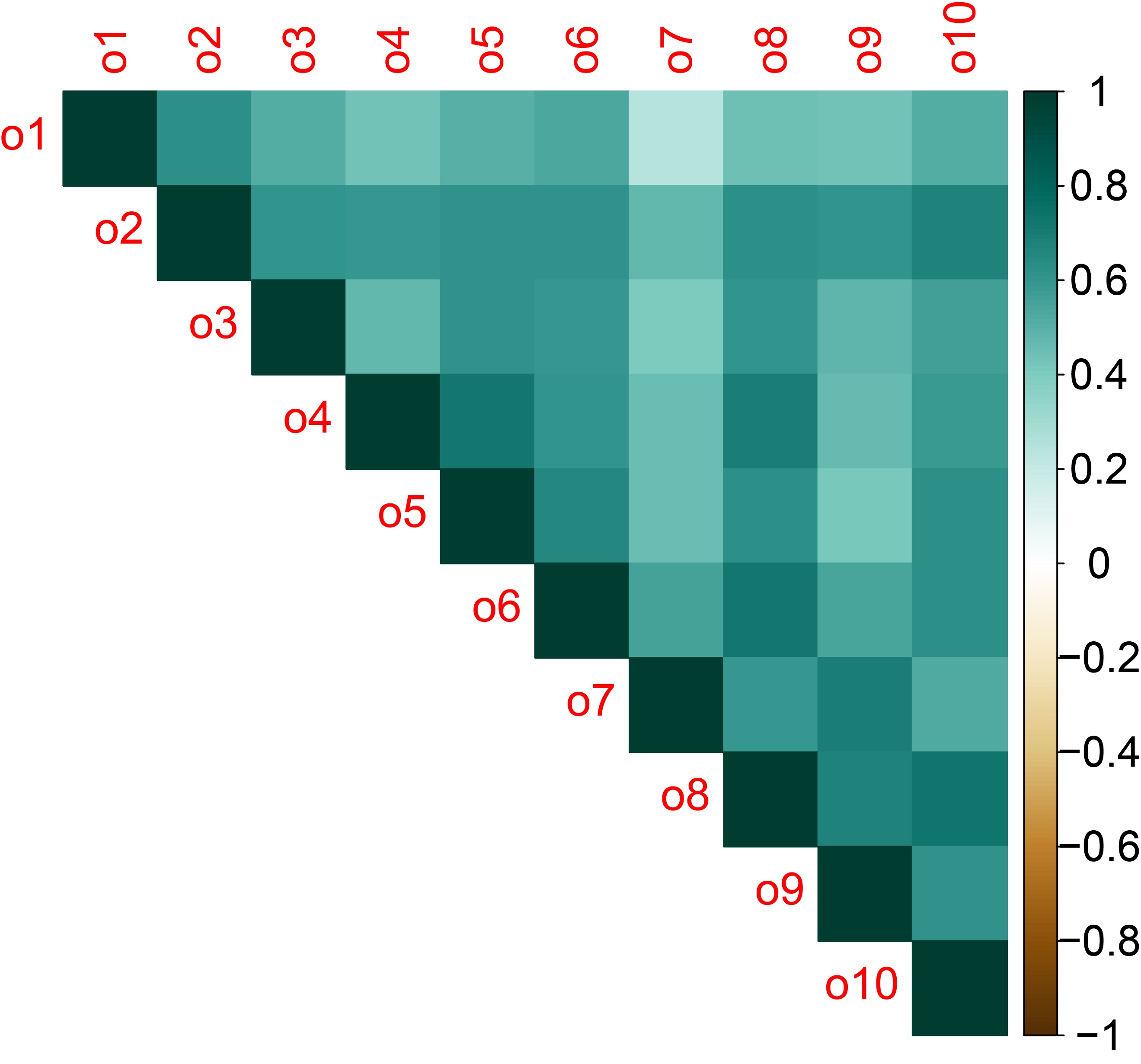
**

**Figure S4-1**. **Heatmap illustrating the item-item correlation of MSI-BPD**. Correlations were computed as polychoric correlations, which estimate the association between latent continuous variables underlying the observed binary item responses. All correlations are statistically significant (*p* ≤ 0.05)

**3. Confirmatory factor analysis**

**
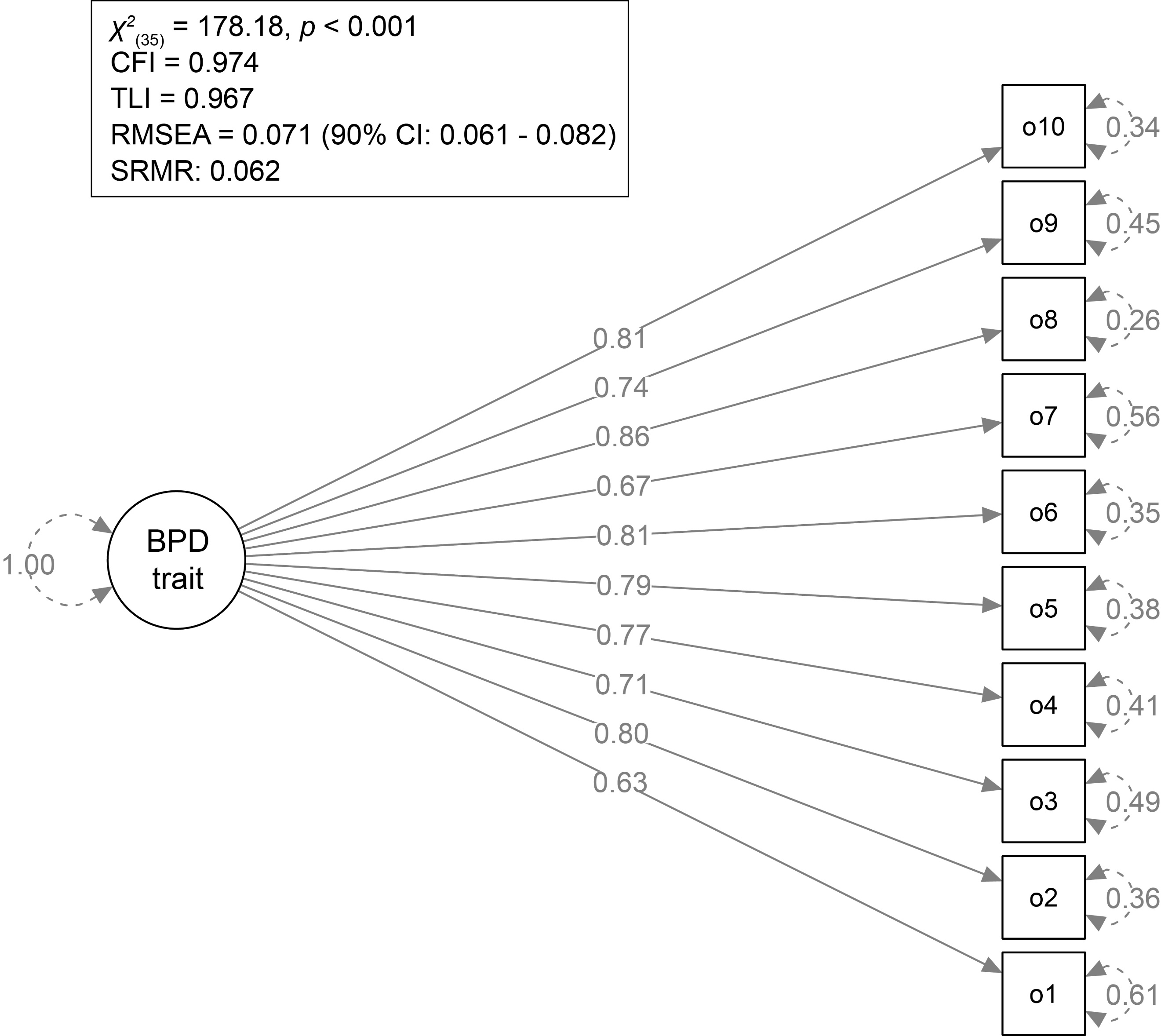
**

**Figure S4-2**. **Confirmatory factor analysis (CFA) of borderline personality trait (BPD trait)**, A one-factor model was fitted to items o1–10. Model fit was good overall. Standardized factor loadings were moderate to strong (0.63–0.86) and all were statistically significant (*p* < .001), supporting a unidimensional latent construct. CFI: Comparative Fit Index, TLI: Tucker–Lewis Index, RMSEA: Root Mean Square Error of Approximation, SRMR: Standardized Root Mean Square Residual.

**Measurement invariance analysis:** Configural, metric, and scalar invariance across groups (gender and age) were tested, and practical changes were evaluated primarily using changes in fit indices (ΔCFI, ΔTLI, ΔRMSEA, ΔSRMR). For models estimated with Weighted least squares mean and variance adjusted (WLSMV) method, robust CFI, TLI, RMSEA and SRMR were used.

*** Gender groups were male and female (Tables S4-1 and S4-2).**

**Table S4-1.** Measurement invariance across gender: Model fit indices (configural, metric, scalar)

| **Model** | **Constraints** | **df** | **χ²***** | **CFI** | **TLI** | **RMSEA** | **SRMR** |
| --- | --- | --- | --- | --- | --- | --- | --- |
| Configural | Same factor structure across gender | 70 | 128.69 | 0.882 | 0.849 | 0.149 | 0.069 |
| Metric | + equal factor loadings | 79 | 253.22 | 0.849 | 0.829 | 0.159 | 0.092 |
| Partial Metric** | + equal factor loadings (5 items only) | 74 | 138.53 | 0.879 | 0.853 | 0.147 | 0.072 |
| Partial Scalar** | + equal factor loadings + thresholds (5 items only) | 83 | 148.15 | 0.889 | 0.879 | 0.147 | 0.072 |

** The “χ²” column contains standard test statistics.*

*** Full metric and scalar invariance were not supported. Partial invariance models were tested by freeing factor loadings and thresholds for items o1, o3, o6, o7, and o9. Items o2, o4, o5, o8, and o10 remained constrained equal across gender groups.*

**Table S4-2**. Measurement invariance across gender: Scaled χ² difference tests and changes in fit indices (ΔCFI, ΔTLI, ΔRMSEA, ΔSRMR)

| **Comparison** | **Δdf** | **Δ χ²** | ***p*** | **ΔCFI** | **ΔTLI** | **ΔRMSEA** | **ΔSRMR** |
| --- | --- | --- | --- | --- | --- | --- | --- |
| Metric vs. Configural | 9 | 64.59 | <0.001 | -0.033 | -0.020 | +0.010 | +0.023 |
| Partial Metric vs. Configural | 4 | 6.47 | 0.166 | -0.003 | +0.004 | -0.002 | +0.003 |
| Partial Scalar vs. Partial Metric | 9 | 14.4 | 0.109 | +0.010 | +0.026 | 0.000 | 0.000 |

Although full metric invariance was not supported (Δχ² = 64.59, *p* < 0.001), partial invariance testing was conducted by freeing items o1, o3, o6, o7, and o9 based on modification indices, while constraining items o2, o4, o5, o8, and o10 equal across gender groups. Partial metric invariance was well-supported with non-significant deterioration from the configural model (Δχ² = 6.47, *p* = 0.166) and minimal changes in practical fit indices (ΔCFI = −0.003; ΔTLI = 0.004; ΔRMSEA = −0.002; ΔSRMR = 0.003). Partial scalar invariance was equally supported (Δχ² = 14.4, *p* = 0.109) with acceptable fit index changes (ΔCFI = 0.010; ΔTLI = 0.026; ΔRMSEA = 0.000; ΔSRMR = 0.000). With 50% of items demonstrating invariance across gender groups, the MSI–BPD scale supports meaningful comparisons of factor means between male and female participants.

*** Age groups were “< 15 years” and “≥ 15 years” (Tables S4-3 and S4-4).**

**Table S4-3.** Measurement invariance across age: Model fit indices (configural, metric, scalar)

| **Model** | **Constraints** | **df** | **χ²***** | **CFI** | **TLI** | **RMSEA** | **SRMR** |
| --- | --- | --- | --- | --- | --- | --- | --- |
| Configural | Same factor structure across gender | 70 | 137.75 | 0.870 | 0.832 | 0.161 | 0.068 |
| Metric | + equal factor loadings | 79 | 196.06 | 0.866 | 0.847 | 0.154 | 0.078 |
| Partial Metric** | + equal factor loadings (6 items ony) | 76 | 140.42 | 0.874 | 0.851 | 0.152 | 0.069 |
| Partial Scalar** | + equal factor loadings + thresholds (6 items only) | 85 | 149.42 | 0.883 | 0.876 | 0.152 | 0.069 |

** The “χ²” column contains standard test statistics.*

** Full metric and scalar invariance were not supported. Partial invariance models were tested by freeing factor loadings and thresholds for items o4, o6, o7, and o9. Items o1, o2, o3, o5, o8, and o10 remained constrained equal across age groups.*

**Table S4-4**. Measurement invariance across age: Scaled χ² difference tests and changes in fit indices (ΔCFI, ΔTLI, ΔRMSEA, ΔSRMR)

| **Comparison** | **Δdf** | **Δ χ²** | ***p*** | **ΔCFI** | **ΔTLI** | **ΔRMSEA** | **ΔSRMR** |
| --- | --- | --- | --- | --- | --- | --- | --- |
| Metric vs. Configural | 9 | 30.00 | <0.001 | -0.004 | 0.015 | -0.007 | 0.010 |
| Partial Metric vs. Configural* | 6 | 1.70 | 0.945 | 0.004 | 0.019 | -0.009 | 0.001 |
| Partial Scalar vs. Partial Metric* | 9 | 13.96 | 0.124 | 0.009 | 0.025 | 0.000 | 0.000 |

Although full metric invariance was not supported (Δχ² = 30.00, *p* < 0.001), partial invariance testing was conducted by freeing items o4, o6, o7, and o9 based on modification indices, while constraining items o1, o2, o3, o5, o8, and o10 equal across age groups. Partial metric invariance was well-supported with non-significant deterioration from the configural model (Δχ² = 1.70, *p* = 0.945) and favorable changes in practical fit indices (ΔCFI = 0.004; ΔTLI = 0.019; ΔRMSEA = -0.009; ΔSRMR = 0.001). Partial scalar invariance was equally supported (Δχ² = 13.96, *p* = 0.124) with acceptable fit index changes (ΔCFI = 0.009; ΔTLI = 0.025; ΔRMSEA = 0.000; ΔSRMR = 0.000). With 60% of items demonstrating invariance across age groups, the MSI-BPD scale supports meaningful comparisons of factor means between younger (< 15 years) and older (≥ 15 years) participants.

**4. Internal inter-item consistence analysis:** Cronbach’s α = 0.86 (Good)
