# Supplementary material for: Assessing the prevalence, characteristics and psychosocial correlates of nonsuicidal self-injury among Vietnamese adolescent psychiatric outpatients: a cross-sectional study
Source: Front Psychiatry. 2026 Feb 18;17:1699844. doi: 10.3389/fpsyt.2026.1699844 (PMC12957150; doi:10.3389/fpsyt.2026.1699844)
Supplement: Supplementary file 5 [file Table5.docx]

*Supplementary material 5:*

**The Alexian Brothers Urge to Self-Injure (ABUSI) Scale validation**

**1. The Content Validity Index (CVI):**

- Item-level CVI (I-CVI) = 1
- Scale-level CVI (S-SVI) = 1

**2. Item-item correlation of the ABUSI scale**


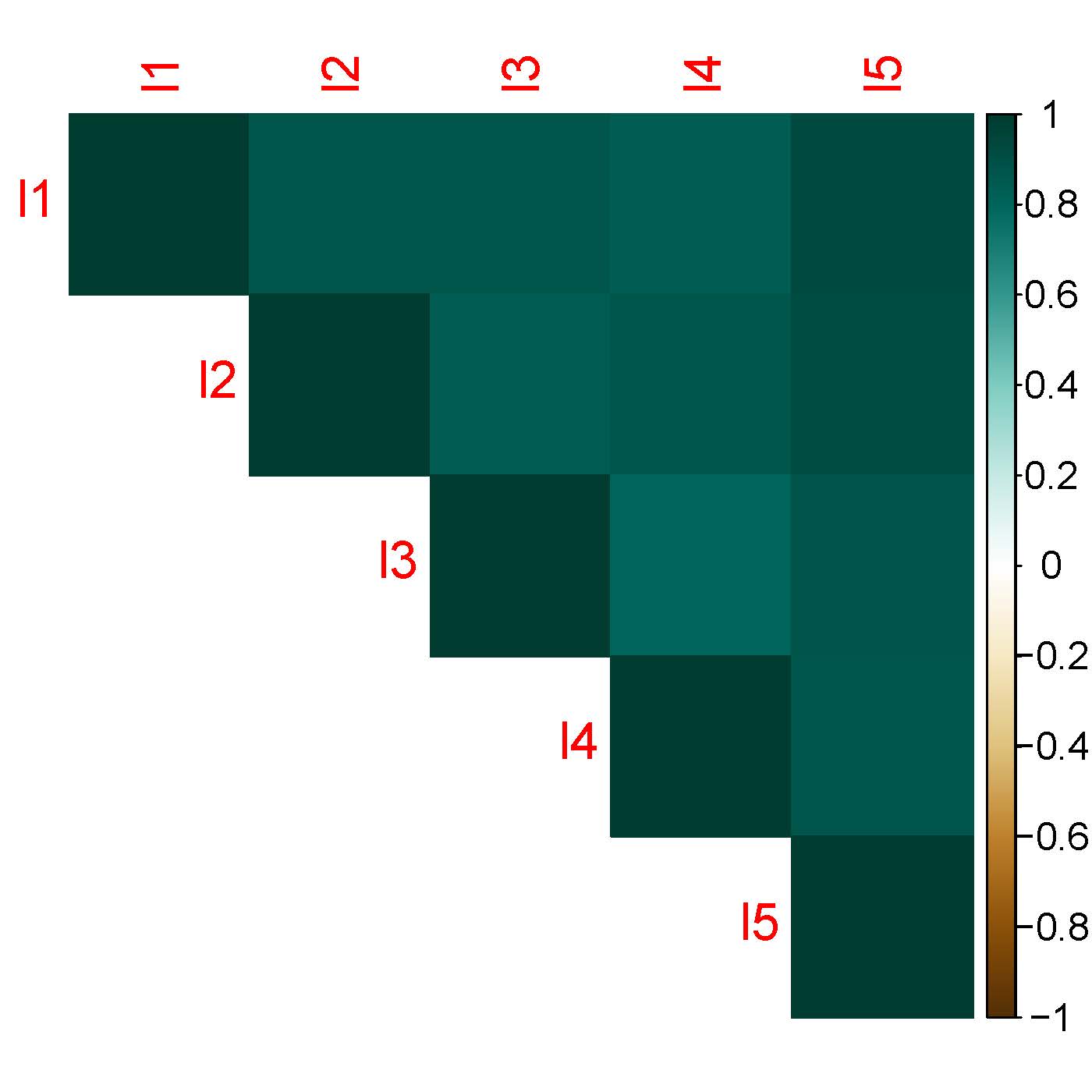


**Figure S5-1**. **Heatmap illustrating the item-item correlation of ABUSI scale**. Correlations were computed as polychoric correlations, which estimate the association between latent continuous variables underlying the observed ordinal item responses. All correlations are statistically significant (*p* ≤ 0.05)

**3. Confirmatory factor analysis**


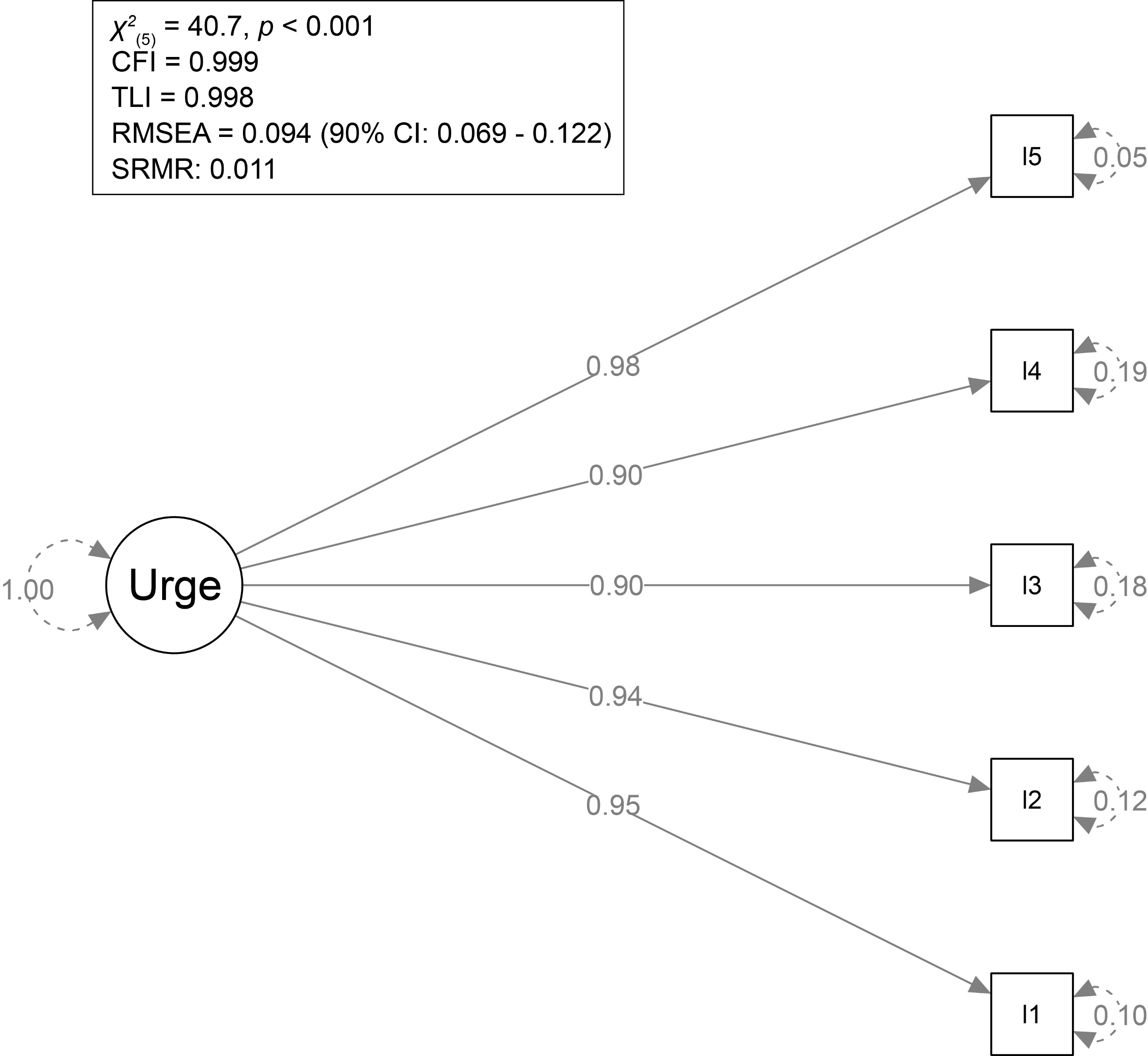


**Figure S5-2**. **Confirmatory factor analysis (CFA) of urge to self-injure (Urge)**, A one-factor model was fitted to items l1–5. Model fit was excellent overall. Standardized factor loadings were very strong (0.90–0.98) and all were statistically significant (*p* < 0.001). CFI: Comparative Fit Index, TLI: Tucker–Lewis Index, RMSEA: Root Mean Square Error of Approximation, SRMR: Standardized Root Mean Square Residual.

**Measurement invariance analysis:** Configural, metric, and scalar invariance across groups (gender and age) were tested, and practical changes were evaluated primarily using changes in fit indices (ΔCFI, ΔTLI, ΔRMSEA, ΔSRMR). For models estimated with Weighted least squares mean and variance adjusted (WLSMV) method, robust CFI, TLI, RMSEA and SRMR were used.

*** Gender groups were male and female (Tables S5-1 and S5-2).**

**Table S5-1.** Measurement invariance across gender: Model fit indices (configural, metric, scalar)

| **Model** | **Constraints** | **df** | **χ²*** | **CFI** | **TLI** | **RMSEA** | **SRMR** |
| --- | --- | --- | --- | --- | --- | --- | --- |
| Configural | Same factor structure across gender | 10 | 11.419 | 0.993 | 0.985 | 0.102 | 0.011 |
| Metric | + equal factor loadings | 14 | 27.576 | 0.981 | 0.972 | 0.141 | 0.017 |
| Partial Metric** | + equal factor loadings (3 items only) | 12 | 17.169 | 0.990 | 0.983 | 0.109 | 0.014 |
| Partial Scalar** | + equal factor loadings + thresholds (3 items only) | 31 | 20.851 | 1.000 | 1.007 | 0.122 | 0.011 |

** The “χ²” column contains standard test statistics.*

*** Full metric and scalar invariance were not supported. Partial invariance models were tested by freeing factor loadings and thresholds for items l3 and l5. Items l1, l2, and l4 remained constrained equal across gender groups.*

**Table S5-2**. Measurement invariance across gender: Scaled χ² difference tests and changes in fit indices (ΔCFI, ΔTLI, ΔRMSEA, ΔSRMR)

| **Comparison** | **Δdf** | **Δ χ²** | ***p*** | **ΔCFI** | **ΔTLI** | **ΔRMSEA** | **ΔSRMR** |
| --- | --- | --- | --- | --- | --- | --- | --- |
| Metric vs. Configural | 4 | 15.99 | 0.003 | −0.012 | −0.013 | 0.039 | +0.006 |
| Partial Metric vs. Configural | 2 | 5.84 | 0.054 | -0.003 | -0.002 | 0.007 | 0.003 |
| Partial Scalar vs. Partial Metric | 19 | 13.44 | 0.816 | 0.010 | 0.024 | 0.013 | 0.003 |

Although full metric invariance was not supported (Δχ² = 15.99, *p* = 0.003), partial invariance testing was conducted by freeing items l3 and l5 based on modification indices, while constraining items l1, l2, and l4 equal across gender groups. Partial metric invariance was well-supported with non-significant deterioration from the configural model (Δχ² = 5.84, *p* = 0.054) and minimal changes in practical fit indices (ΔCFI = -0.003; ΔTLI = -0.002; ΔRMSEA = 0.007; ΔSRMR = 0.003). Partial scalar invariance was equally supported (Δχ² = 13.44, *p* = 0.816) with acceptable fit index changes (ΔCFI = 0.010; ΔTLI = 0.024; ΔRMSEA = 0.013; ΔSRMR = -0.003). With 60% of items demonstrating invariance across gender groups, the scale supports meaningful comparisons of factor means between male and female participants.

*** Age groups were “< 15 years” and “≥ 15 years” (Tables S5-3 and S5-4).**

**Table S5-3.** Measurement invariance across age: Model fit indices (configural, metric, scalar)

| **Model** | **Constraints** | **df** | **χ²*** | **CFI** | **TLI** | **RMSEA** | **SRMR** |
| --- | --- | --- | --- | --- | --- | --- | --- |
| Configural | Same factor structure across age | 10 | 10.580 | 0.991 | 0.983 | 0.112 | 0.011 |
| Metric | + equal factor loadings | 14 | 12.064 | 0.993 | 0.990 | 0.085 | 0.012 |
| Scalar | + equal loadings + equal thresholds | 33 | 21.533 | 1.000 | 1.001 | 0.118 | 0.011 |

** The “χ²” column contains standard test statistics.*

**Table S5-4**. Measurement invariance across age: Scaled χ² difference tests and changes in fit indices (ΔCFI, ΔTLI, ΔRMSEA, ΔSRMR)

| **Comparison** | **Δdf** | **Δ χ²** | ***p*** | **ΔCFI** | **ΔTLI** | **ΔRMSEA** | **ΔSRMR** |
| --- | --- | --- | --- | --- | --- | --- | --- |
| Metric vs. Configural | 4 | 1.897 | 0.755 | 0.002 | 0.007 | -0.027 | 0.001 |
| Scalar vs. Metric | 19 | 27.499 | 0.094 | 0.007 | 0.011 | 0.033 | -0.001 |

The Scaled χ² difference tests supported invariance across age groups, indicating no statistically significant decrement in model fit when moving from the configural to the metric model (Δχ² = 1.897, Δdf = 4, *p* = 0.755) or from the metric to the scalar model (Δχ² = 27.499, Δdf = 19, *p* = 0.094). Measurement invariance was also evaluated using changes in practical fit indices. These changes were small from the configural to the metric model (ΔCFI = 0.002; ΔTLI = 0.007; ΔRMSEA = −0.027; ΔSRMR = 0.001) and from the metric to the scalar model (ΔCFI = 0.007; ΔTLI = 0.011; ΔRMSEA = 0.033; ΔSRMR = −0.001), supporting overall measurement invariance across age.

**4. Internal inter-item consistence analysis:** Cronbach’s α = 0.94 (Excellent)

**5. Criterion-related validity (ROC/AUC)**


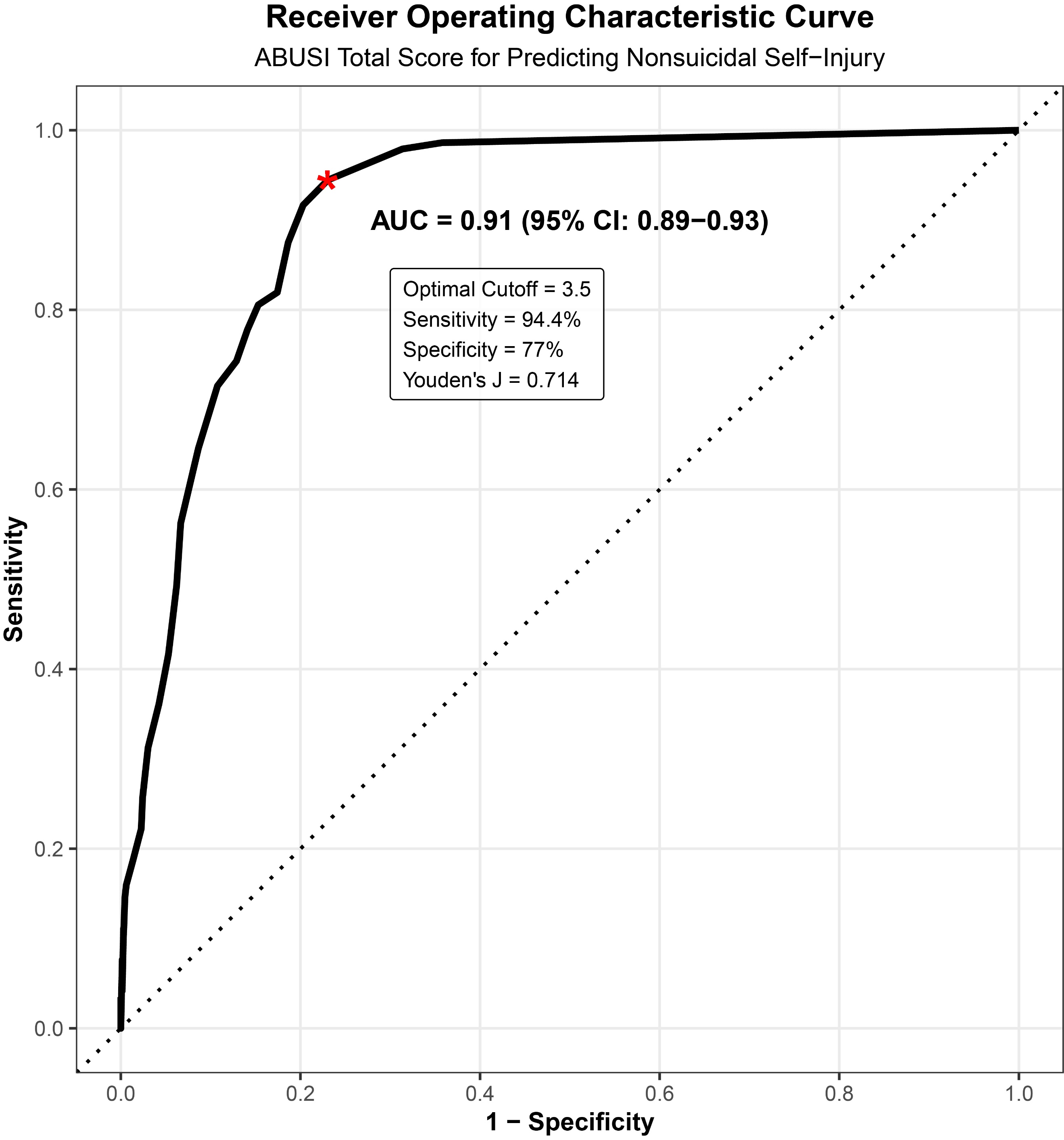


**Figure S5-3. Receiver operating characteristic (ROC) curve.** The ABUSI total score showed excellent discrimination for predicting nonsuicidal self-injury (AUC = 0.91, 95% CI [0.89, 0.93]). The Youden-optimal cutoff (ABUSI ≥ 3.5) yielded sensitivity = 94.4% and specificity = 77.0% (Youden’s J = 0.714), supporting its use as a screening threshold for identifying individuals at elevated likelihood of NSSI. AUC: Area under curve.
