# Supplementary material for: Assessing the prevalence, characteristics and psychosocial correlates of nonsuicidal self-injury among Vietnamese adolescent psychiatric outpatients: a cross-sectional study
Source: Front Psychiatry. 2026 Feb 18;17:1699844. doi: 10.3389/fpsyt.2026.1699844 (PMC12957150; doi:10.3389/fpsyt.2026.1699844)
Supplement: Supplementary file 6 [file Table6.docx]

*Supplemental material 6:*

**Conceptual framework of factors associated with NSSI DSM-5 and multicollinearity assessment**

**
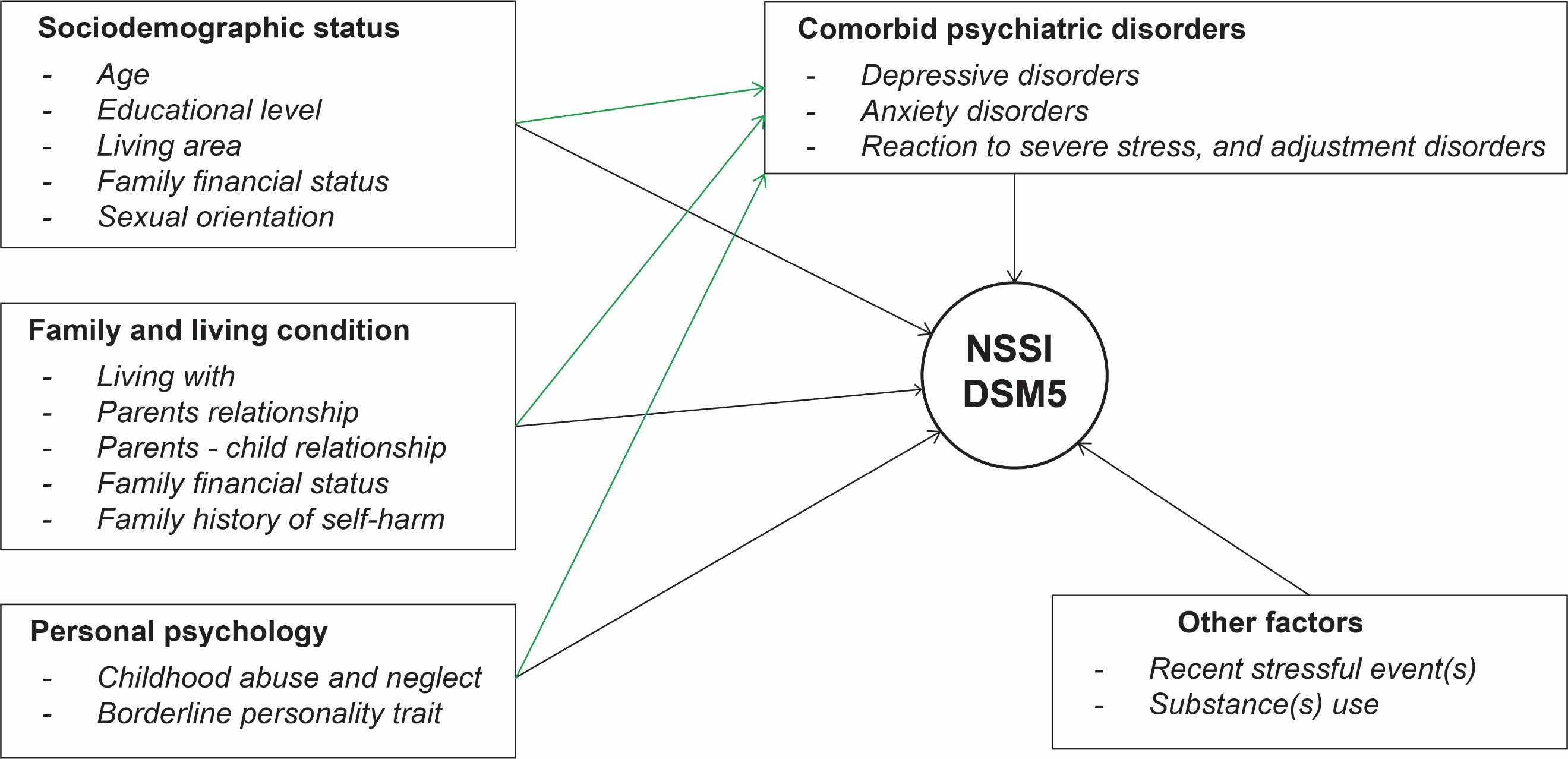
**

**Figure S6-1. Conceptual framework of factors associated with NSSI DSM5**

**
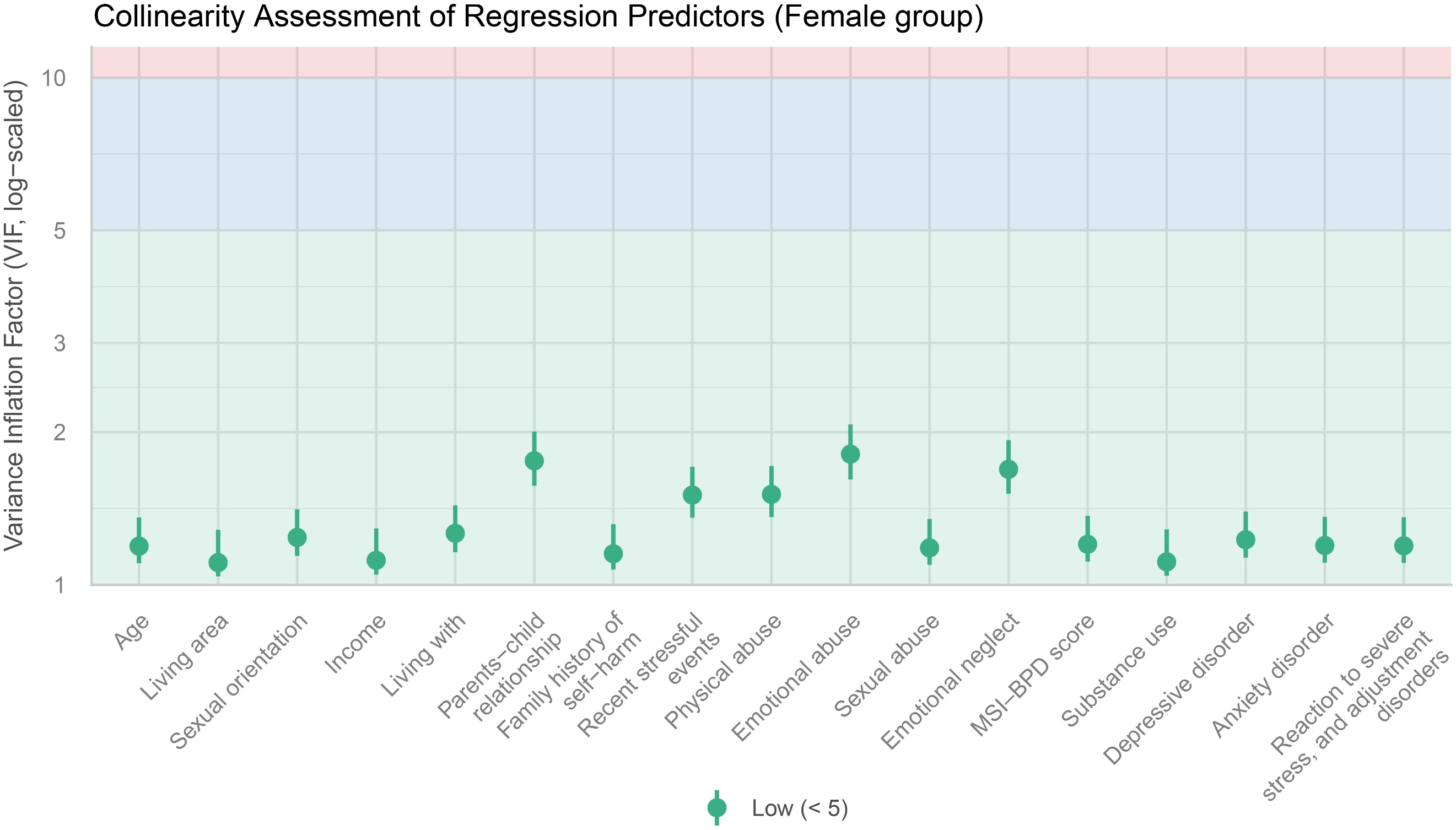
**

**Figure S6-2. Collinearity assessment of regression predictors of NSSI DSM5, female group**


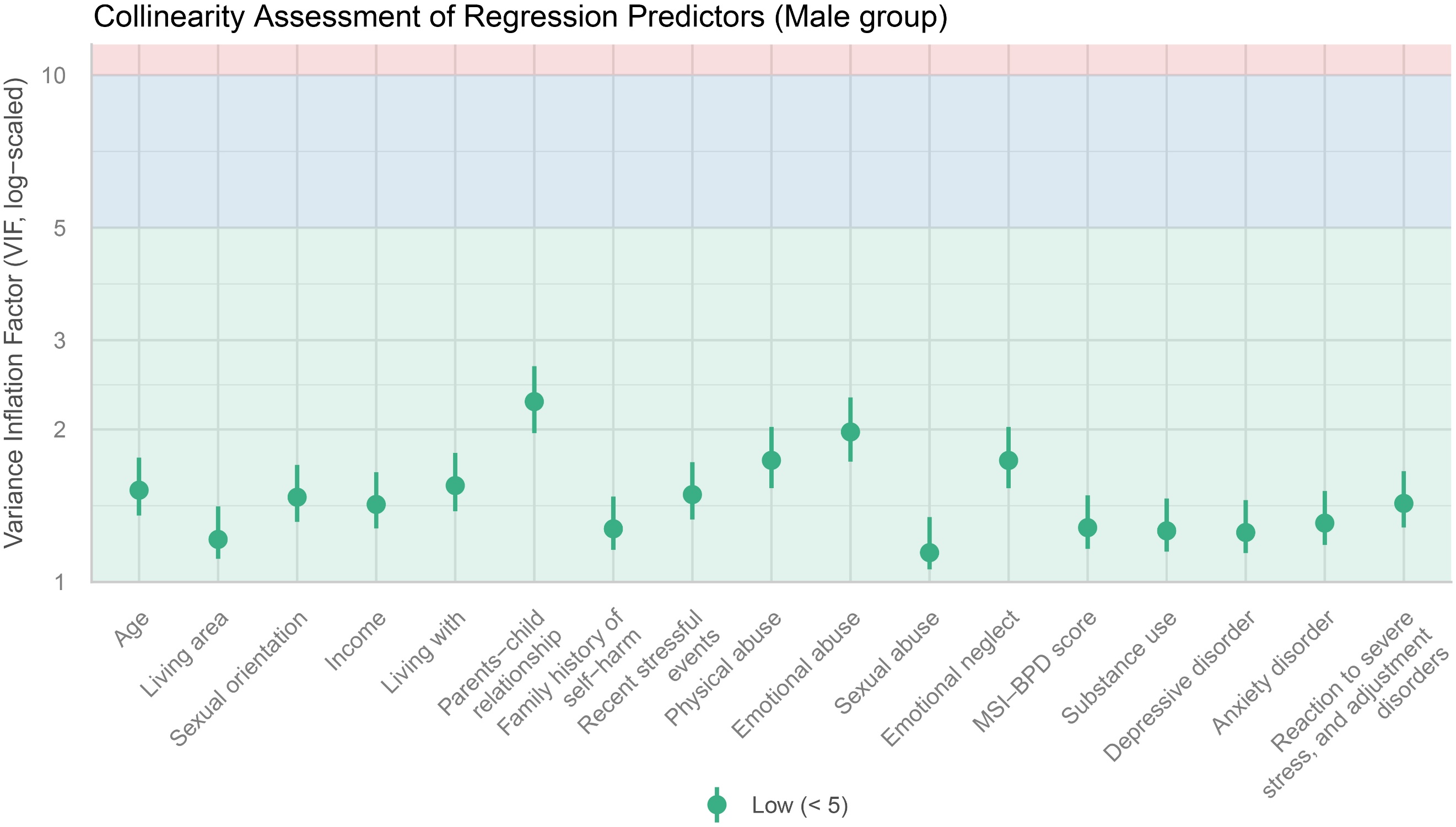


**Figure S6-3. Collinearity assessment of regression predictors of NSSI DSM5, male group**

In the multivariable logistic regression models for both male and female participants, the variable 'Educational level' was excluded due to potential collinearity with “Age“ variable. This is due to the age-range selection (10 – 19 years) in this study. Additionally, the variable “Parents relationships” variabe was excluded from the models due to potential collinearity with “Living with” variable. These decisions were made to address potential multicollinearity concerns and improve the stability and interpretability of the regression estimates. To account for potential between-center differences, “Institution” variable was also included in all multivariable models as a categorical fixed effect (indicator variables) but not visualized in **Figure S6-2** and **S6-3**
