# Supplementary material for: Assessing the prevalence, characteristics and psychosocial correlates of nonsuicidal self-injury among Vietnamese adolescent psychiatric outpatients: a cross-sectional study
Source: Front Psychiatry. 2026 Feb 18;17:1699844. doi: 10.3389/fpsyt.2026.1699844 (PMC12957150; doi:10.3389/fpsyt.2026.1699844)
Supplement: Supplementary file 7 [file Table7.docx]

*Supplementary material 7:*

**Adolescent Self-Harm Safety Protocol**

**Step 1. Risk assessment & identification**

Start every relevant visit/exam with a safety screen.

1. Conduct a clinical assessment to identify elevated risk of self-harm.
2. Evaluate for:
   - Non-suicidal self-injury (NSSI), and/or
   - Suicidal ideation and/or intent
3. Document all findings in the clinical record.

**Step 2. Immediate response (when elevated risk is detected)**

Move immediately into a higher-level safety workflow.

1. Clinician performs an immediate risk evaluation.
2. Discuss findings and recommended next steps with:
   - The adolescent
   - The legal guardian (when present)

**Step 3. Decision-making framework**

Use the appropriate pathway based on intent and acuity.

**3.1. NSSI without suicidal intent**

1. Hold a shared discussion about options and safety needs.
2. Hospitalization requires agreement from both:
   - Adolescent AND
   - Guardian
3. If both agree: proceed with hospitalization per plan.

**3.2. suicidal ideation/intent with acute risk**

1. Clinician makes a clinical recommendation for urgent action.
2. Guardian holds decision-making authority.
3. Follow applicable clinical and legal standards.
4. Arrange urgent psychiatric evaluation and/or hospitalization as clinically indicated.

**Step 4. If recommended hospitalization is refused (adolescent + guardian decline)**

Provide immediate crisis guidance and clear instructions.

1. Provide written crisis help-seeking instructions.
2. Provide verbal crisis help-seeking instructions.
3. Include:
   - Emergency contact numbers
   - Clinic contact information
4. Instruct adolescent to contact the clinic immediately if feeling unsafe.

**Step 5. Guardian Unavailable Protocol**

Use one of the following routes depending on contact feasibility.

**5.1. If guardian is unavailable (but contactable)**

1. Clinical team attempts to contact family/guardian.
2. Communicate safety concerns.
3. Coordinate further care arrangements.

**5.2. If Adolescent Refuses to Provide Guardian Contact Information**

1. Provide written crisis help-seeking instructions.
2. Provide verbal crisis help-seeking instructions.
3. Include:
   - Emergency contact numbers
   - Clinic contact information
4. Instruct adolescent to contact the clinic immediately if feeling unsafe.

**Step 6. Documentation & Compliance**

Close the loop with required documentation and policy adherence.

1. Follow institutional policies.
2. Observe limits of confidentiality in acute safety-risk situations.
3. Document all procedures in the clinical record.
4. Apply protocol to adolescents ages 10–19 per clinical policy.
